# Mixed chloridometallate(ii) ionic liquids with tunable color and optical response for potential ammonia sensors†

**DOI:** 10.1039/d2ra05581c

**Published:** 2022-12-08

**Authors:** Karsten Behrens, Christian Balischewski, Eric Sperlich, Antonia Menski, Ruth Fabiola Balderas-Valadez, Claudia Pacholski, Christina Günter, Susanne Lubahn, Alexandra Kelling, Andreas Taubert

**Affiliations:** Institute of Chemistry, University of Potsdam Karl-Liebknecht-Strasse 24-25 D-14476 Potsdam Germany ataubert@uni-potsdam.de; Institute of Geosciences, University of Potsdam Karl-Liebknecht-Strasse 24-25 D-14476 Potsdam Germany

## Abstract

Eight d-metal-containing *N*-butylpyridinium ionic liquids (ILs) with the nominal composition (C_4_Py)_2_[Ni_0.5_M_0.5_Cl_4_] or (C_4_Py)_2_[Zn_0.5_M_0.5_Cl_4_] (M = Cu, Co, Mn, Ni, Zn; C_4_Py = *N*-butylpyridinium) were synthesized, characterized, and investigated for their optical properties. Single crystal and powder X-ray analysis shows that the compounds are isostructural to existing examples based on other d-metal ions. Inductively coupled plasma optical emission spectroscopy measurements confirm that the metal/metal ratio is around 50 : 50. UV-Vis spectroscopy shows that the optical absorption can be tuned by selection of the constituent metals. Moreover, the compounds can act as an optical sensor for the detection of gases such as ammonia as demonstrated *via* a simple prototype setup.

## Abstract 2

Eight d-metal-containing *N*-butylpyridinium-based ionic liquids (ILs) were synthesized, characterized, and investigated for their optical properties.

## Graphical abstract

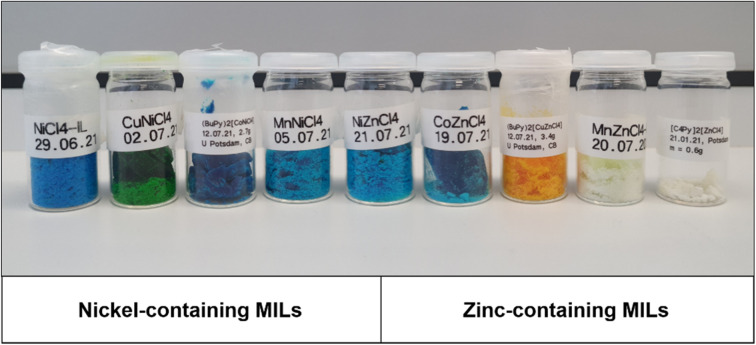

## Introduction

Ionic Liquids (ILs) have technologically relevant properties and have been explored for their use as solvents,^1–3^ in catalysis,^4^ separation,^5^ photoluminescence,^6^ drug delivery,^7^ and electrochemistry.^8–10^ One of the main features of ILs is the fact that their properties can be adjusted by carefully selecting the constituting anions and cations.^11^ Consequently, ILs can be tuned for their melting points, thermal and chemical stability, volatility, (non-)flammability, ionic conductivity, or electrochemical stability windows.^12–16^

Metal containing ILs (MILs) built around the MX_4_^2−^ anion (M = Fe, Co, Ni, Cu, Zn, Al, In, Au, …; X = Cl, Br, I, SCN, …) have shown an especially widespread diversity of properties. This is due to the fact that the simple exchange of a metal ion for another one leads to significant changes in the optical, electronic, magnetic, electrochemical, or catalytic properties.^17–19^ Moreover, also the exchange of the X^−^ anion directly affects the properties of the resulting IL.^20–23^

One of the striking features of these tetrahalidometallate compounds (both their complexes and their ILs) is their intense color and color change upon exchange of a cation or ligand in the MX_4_^2−^ unit. MX_4_^2−^ based compounds also show a strong solvato- or vapochromism,^24,25^ suggesting applications in gas or solvent sensing. Moreover, the reaction of gases like ammonia with MX_4_^2−^ can be exploited for both sensing and gas separation.^26,27^

Extending this concept, ILs combining two or more metals in one IL may be of interest for optical sensing because the combination of different metals provides access to different optical windows. This can lead to improved sensitivities or selectivities for different solvent or gas molecules in the detection process. Indeed, Balischewski *et al.* have recently shown that the combination of two or three metal ions in MX_4_^2−^-based ILs leads to ILs with a range of colors from blue to green to yellow and orange, although no tests on their sensing capabilities have been made so far.^23,28^

The current article extends the previous work in two directions: (1) it expands the pool of mixed d-metal-based ILs to new bimetallic ILs built around Ni(ii) and Zn(ii) and (2) the article also demonstrates that the compounds can be used as the active component in an optical setup for the detection of ammonia gas using a fiber optic probe and a simple IL-coated glass slide.

## Results

**Fig. 1:**
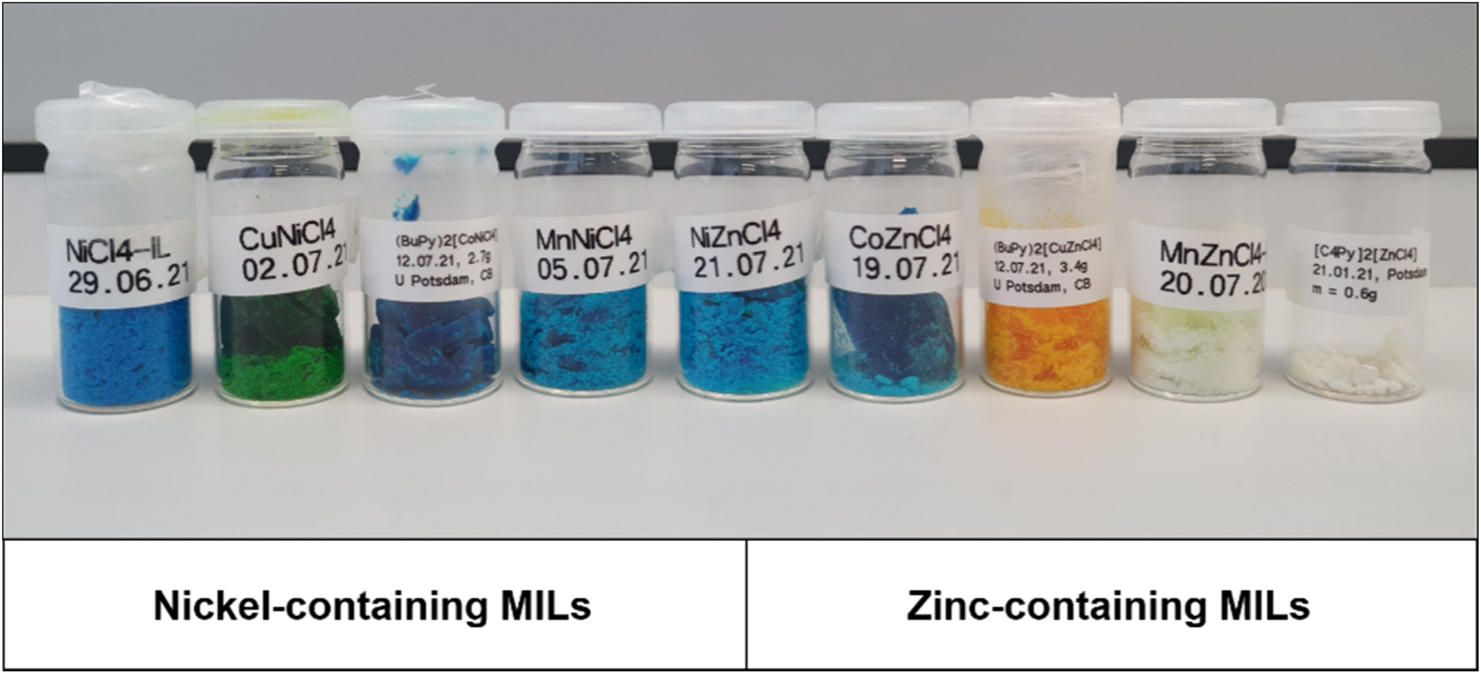
Ni- and Zn-based mixed metal ILs. Nominal composition is always (C_4_Py)_2_[Ni_0.5_M_0.5_Cl_4_] or (C_4_Py)_2_[Zn_0.5_M_0.5_Cl_4_]. For precise metal contents see Table 1 below.

The ILs bis-*N*-butylpyridinium tetrachlorido nickelate(ii), (C_4_Py)_2_[NiCl_4_] and bis-*N*-butylpyridinium tetrachlorido zincate(ii), (C_4_Py)_2_[ZnCl_4_] were synthesized according to published protocols.^22,29^ The new mixed d-metal-based ILs with a nominal composition (C_4_Py)_2_[Ni_0.5_M_0.5_Cl_4_] and (C_4_Py)_2_[Zn_0.5_M_0.5_Cl_4_] (M = Cu, Co, Mn, Ni, Zn) were synthesized by employing appropriate mixtures of the respective metal chlorides using the same protocol. These compounds exhibit a range of colors depending on their composition, Fig. 1.

Table 1 summarizes the ILs investigated in this study. Inductively coupled plasma optical emission spectroscopy (ICP OES) shows that the metal contents and metal : metal ratios are in good agreement with the synthetic protocol. A 50 : 50 metal ratio was the target and in most cases, the ILs exhibit ratios that are very close to this target ratio. Furthermore, some of the ILs exhibit an overall lower metal content than what is expected for a clean and pure IL. This lower total metal content can be assigned to water uptake. For example, IL 2 contains 5.68 and 6.16 wt% of Ni and Cu, respectively. The theoretical values are slightly higher at 6.17 and 6.68 wt% for a 50 : 50 metal ratio. A calculation taking into account the Ni and Cu fractions of 5.36 and 5.80 wt%, respectively, obtained from ICP OES for an exact 50 : 50 ratio and balancing this with water yields a composition of (C_4_Py)_2_[Ni_0.5_Cu_0.5_Cl_4_]·4H_2_O. Overall, ICP OES shows that the IL compositions are very close to their target composition. ICP OES data also indicates that some of the ILs do take up some water, similar to many other ILs.^30–32^

**Table 1:** IL numbering scheme, metal contents from ICP OES (theoretical values in brackets), and metal : metal ratios determined from ICP OES data^a^

IL	Anion	Metal ion content [w%]	Metal : metal ratio from ICP OES
		Measured (calculated)	
1	[NiCl_4_]^2−^	Ni: 11.23 ± 0.15 (12.41)	n/a
2	[Ni_0.5_Cu_0.5_Cl_4_]^2−^	Ni: 5.68 ± 0.58 (6.17)	50 : 50
		Cu: 6.16 ± 0.65 (6.68)	
3	[Ni_0.5_Co_0.5_Cl_4_]^2−^	Ni: 6.69 ± 0.56 (6.20)	50 : 50
		Co: 5.77 ± 0.68 (6.23)	
4	[Ni_0.5_Mn_0.5_Cl_4_]^2−^	Ni: 6.54 ± 0.28 (6.23)	51 : 49
		Mn: 5.62 ± 0.14 (5.83)	
5	[Ni_0.5_Zn_0.5_Cl_4_]^2−^	Ni: 5.99 ± 0.38 (6.16)	49 : 51
		Zn: 6.97 ± 0.33 (6.86)	
6	[Zn_0.5_Cu_0.5_Cl_4_]^2−^	Zn: 6.31 ± 0.48 (6.83)	47 : 53
		Cu: 6.57 ± 0.13 (6.64)	
7	[Zn_0.5_Co_0.5_Cl_4_]^2−^	Zn: 6.85 ± 0.28 (6.86)	52 : 48
		Co: 6.18 ± 0.34 (6.19)	
8	[Zn_0.5_Mn_0.5_Cl_4_]^2−^	Zn: 6.82 ± 0.07 (6.89)	50 : 50
		Mn: 5.58 ± 0.05 (5.79)	

a Target metal : metal ratio from synthesis is 50 : 50 in all cases

Single crystal structure analysis was performed for compounds 1, 3, and 7 at 210 K. Table 2 summarizes the relevant crystallographic data of their crystal structures. All ILs crystallize in the monoclinic space group *P*2_1_/*n*, consistent with earlier data.^28,33^ The asymmetric unit contains four *N*-butylpyridinium cations and two tetrachloridometallate(ii) anions. For compounds 3 and 7, there is a mixed occupation by the two transition metals on both symmetry-independent atomic positions. The modeling of the occupation of these positions in the final structure refinement was done by using two split positions, where the occupation of the atomic position was fixed to 100%. For compound 3, the refinement resulted in an atomic occupation of Co and Ni of 50 : 50 and for compound 7, an atomic occupation of Co and Zn in the ratio 0.46 : 0.54 is obtained.

**Table 2:** Crystallographic data and details of the refinement of ILs 1, 3, and 7

Compounds	(C_4_Py)_2_[NiCl_4_] (1)	(C_4_Py)_2_[Ni_0.5_Co_0.5_Cl_4_] (3)	(C_4_Py)_2_[Zn_0.54_Co_0.46_Cl_4_] (7)
Molecular formula	C_18_H_28_Cl_4_N_2_Ni	C_18_H_28_Cl_4_Co_0.50_N_2_Ni_0.50_	C_18_H_28_Cl_4_Co_0.46_N_2_Zn_0.54_
*M* [g mol^−1^]	472.93	473.04	476.64
Temperature [K]	210	210	210
Crystal system	Monoclinic	Monoclinic	Monoclinic
Space group	*P*2_1_/*n* (14)	*P*2_1_/*n* (14)	*P*2_1_/*n* (14)
*a* [Å]	15.4965(3)	15.3911(4)	15.3694(4)
*b* [Å]	18.4666(4)	18.7389(5)	18.7506(5)
*c* [Å]	16.7917(3)	16.7383(4)	16.7373(4)
*β* [°]	110.529(1)	110.494(2)	110.442(2)
*V* [Å^3^]	4500.08(16)	4522.00(20)	4519.7(2)
*Z*	8	8	8
*D*_calcd_ [g cm^−3^]	1.396	1.390	1.401
*μ* [mm^−1^]	1.34	1.29	1.41
Reflection collected	82 567	157 362	157 574
Independent reflection	7897	7950	7947
Reflections *I* > 2*σ*(*I*)	7041	5903	6229
*R*_int_	0.031	0.054	0.043
No. refined parameters	456	485	529
*R*[*F*^2^ > 2*σ*(*F*^2^)], w*R*(*F*^2^), *S*	0.026, 0.072, 1.03	0.025, 0.060, 0.92	0.023, 0.058, 0.97
Δ*ρ*_max_, Δ*ρ*_min_ [e Å^−3^]	0.90, −0.33	0.60, −0.24	0.66, −0.26
CCDC	2157870	2157871	2157879

**Fig. 2:**
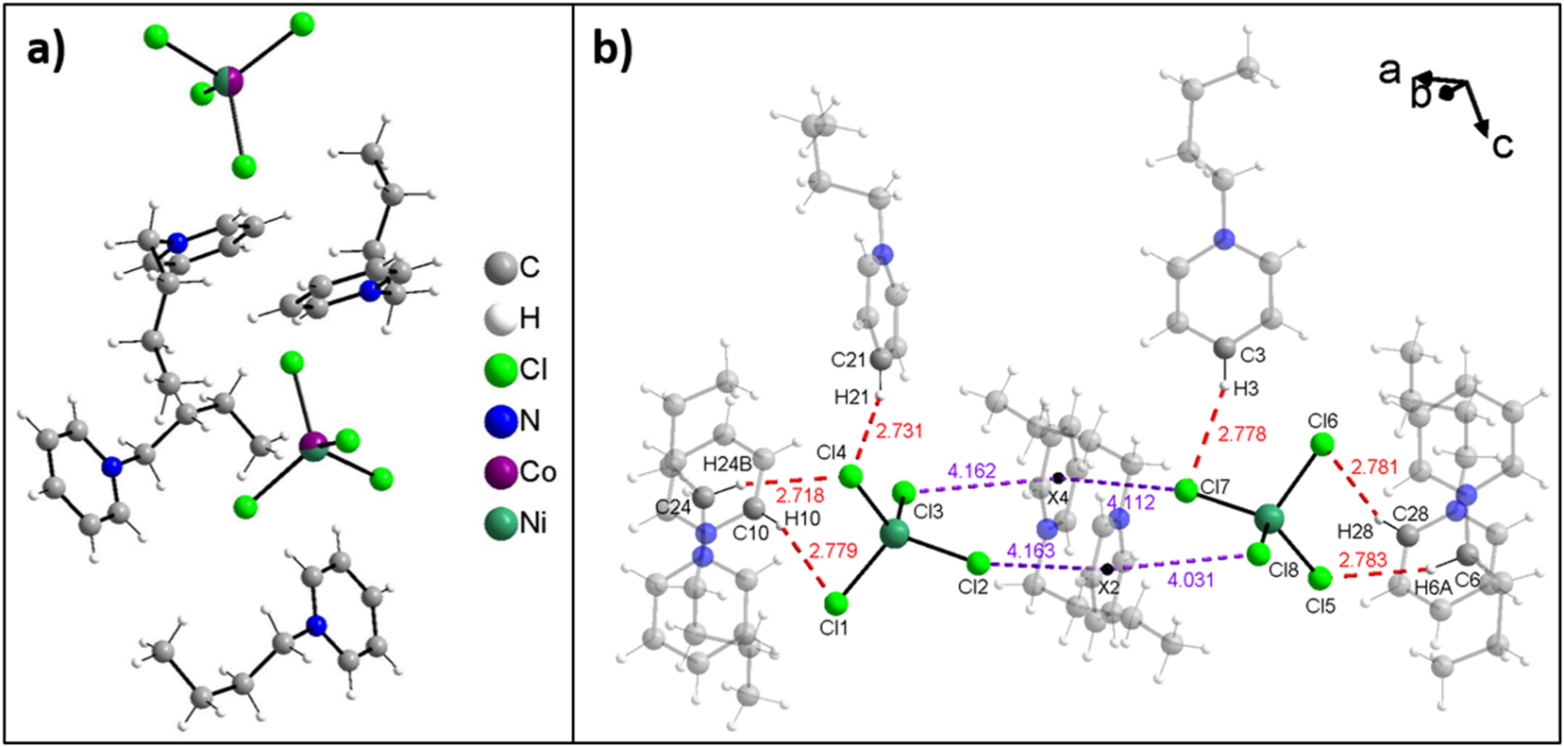
(a) Asymmetric unit of compound 3 with mixed occupation of the transition metal position by Co and Ni and (b) illustration of the C–H⋯Cl hydrogen bonds (red dashed lines) and some anion⋯π interactions (violet dashed line) in compound 1.

Fig. 2a shows the asymmetric unit of compound 3. All structures of the ILs are isotypic and the arrangement of anions and cations in the solid state occurs with the formation of weak C–H⋯Cl hydrogen bridges and anion⋯π interactions. Moreover, no water is observed in the crystal structure, indicating that the water uptake detected from ICP-OES data (Table 1) is reversible.

**Fig. 3:**
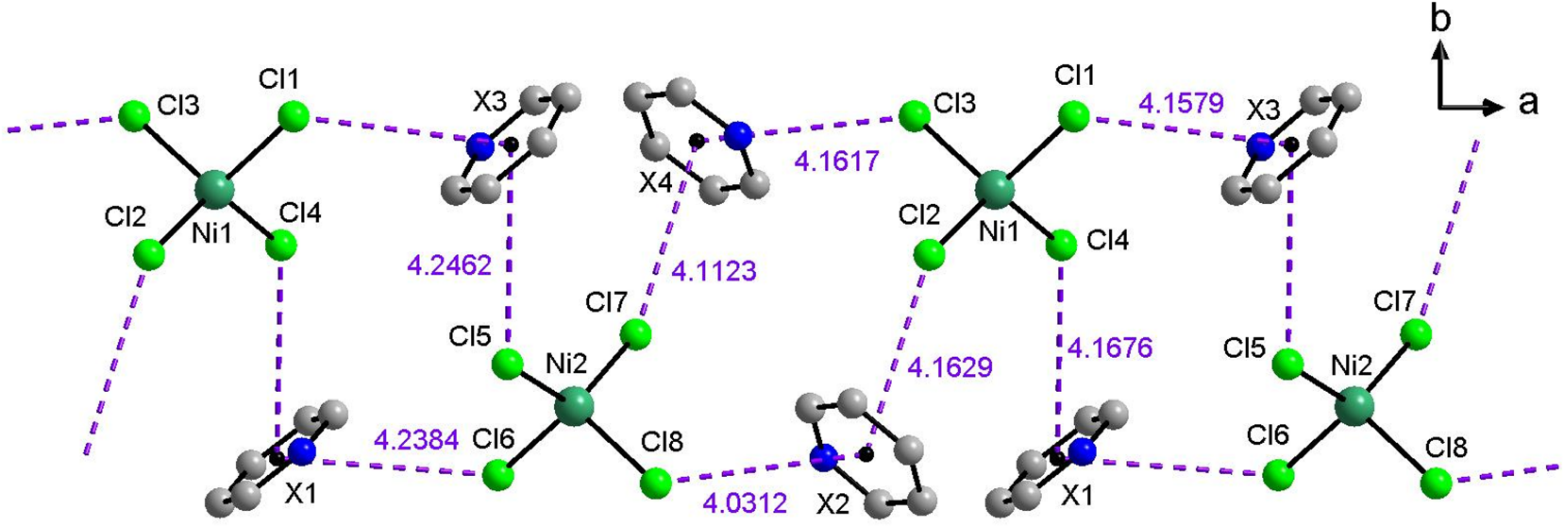
Representation of the anion⋯π interactions (purple dashed lines) in compound 1 leading to intermolecular chains along the crystallographic *a*-axis. X marks the center of the aromatics. Hydrogen atoms and butyl chains are omitted for clarity.

Fig. 2b shows all hydrogen bonds up to a proton-acceptor distances of 2.8 Å for IL 1. Six such symmetry independent hydrogen bonds between the cations and the anions are present. Only two of the four chloride atoms of each anion form hydrogen bonds because the other two chlorides are involved in anion⋯π interactions to the aromatic rings of the cation. Overall, each chloride forms one such anion⋯π interaction and each cation forms two such anion⋯π interactions leading to intermolecular chains along the crystallographic *a*-axis. In each compound eight symmetry independent anion⋯π interactions are present and the distances vary from 4.0 Å to 4.3 Å (Fig. 3). Further figures of the structures, packing, and interactions in the crystals of 1, 3, and 7 are shown in the ESI, Fig. S1–S18, Tables S1–S3.†

**Fig. 4:**
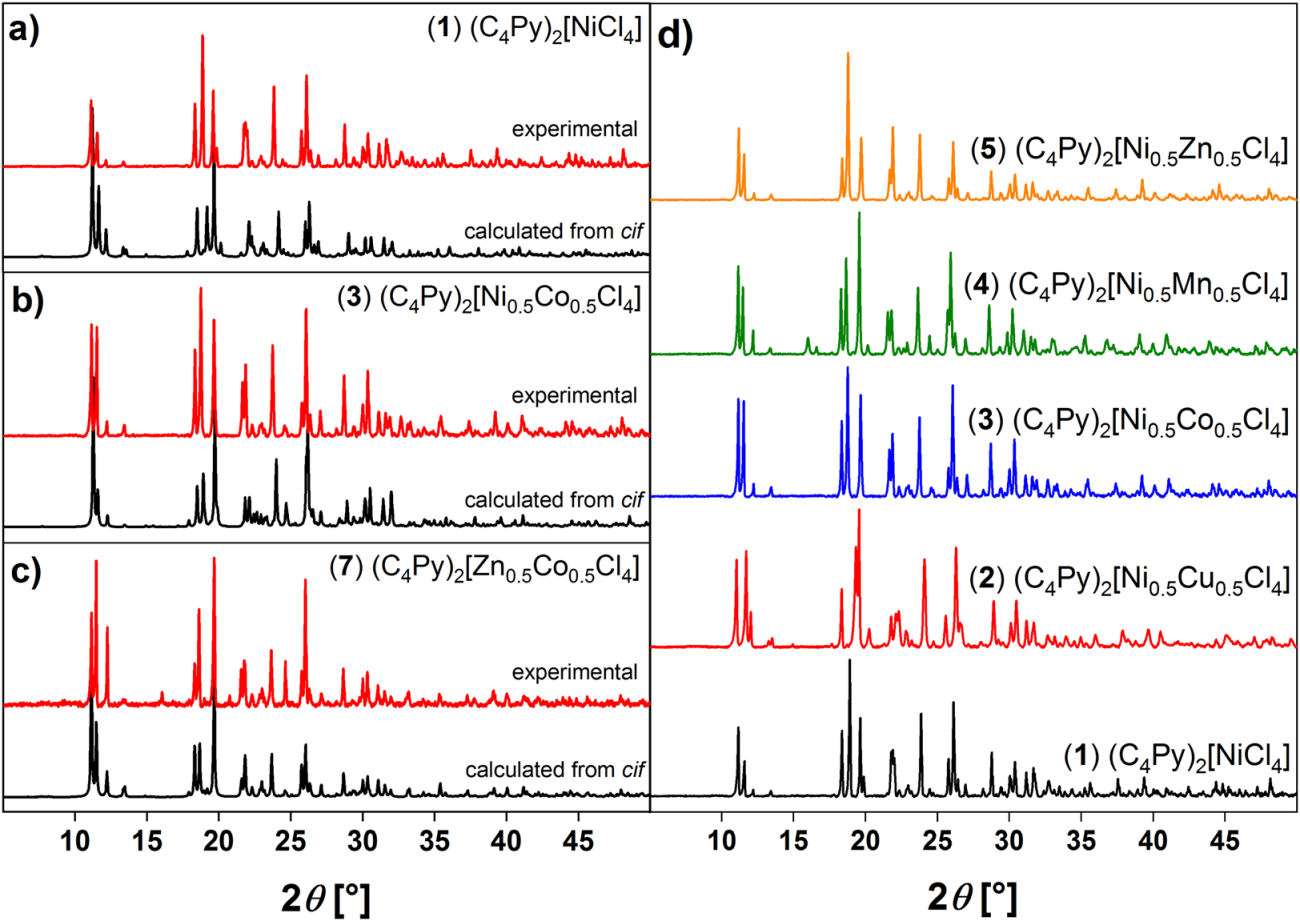
Comparison of XRD patterns calculated from *cif* files (black) and corresponding experimental XRD data (red) of (a) (C_4_Py)_2_[NiCl_4_] (1), (b) (C_4_Py)_2_[Ni_0.5_Co_0.5_Cl_4_] (3), (c) (C_4_Py)_2_[Zn_0.5_Co_0.5_Cl_4_] (7). Panel (d) shows the comparison of the XRD patterns of ILs 1–5. Data are shifted vertically to show all diffractograms clearly; *y*-axis shows reflection intensity in X-ray counts (a.u).

Additional powder X-ray diffraction (XRD) data show that powders obtained from the synthesis have the same crystal structure as the single crystals. Comparison of the XRD patterns with patterns computed from the respective single crystal structures show a very good agreement with the experimental XRD patterns thus confirming identical structures, Fig. 4a–c. All ILs are isostructural to one another and show a relatively good crystallinity (Fig. 4). These data also agree very well with previous data.^28,33^ Fig. S19† also shows that these patterns agree well with those obtained from the compounds based on only one metal.

**Fig. 5:**
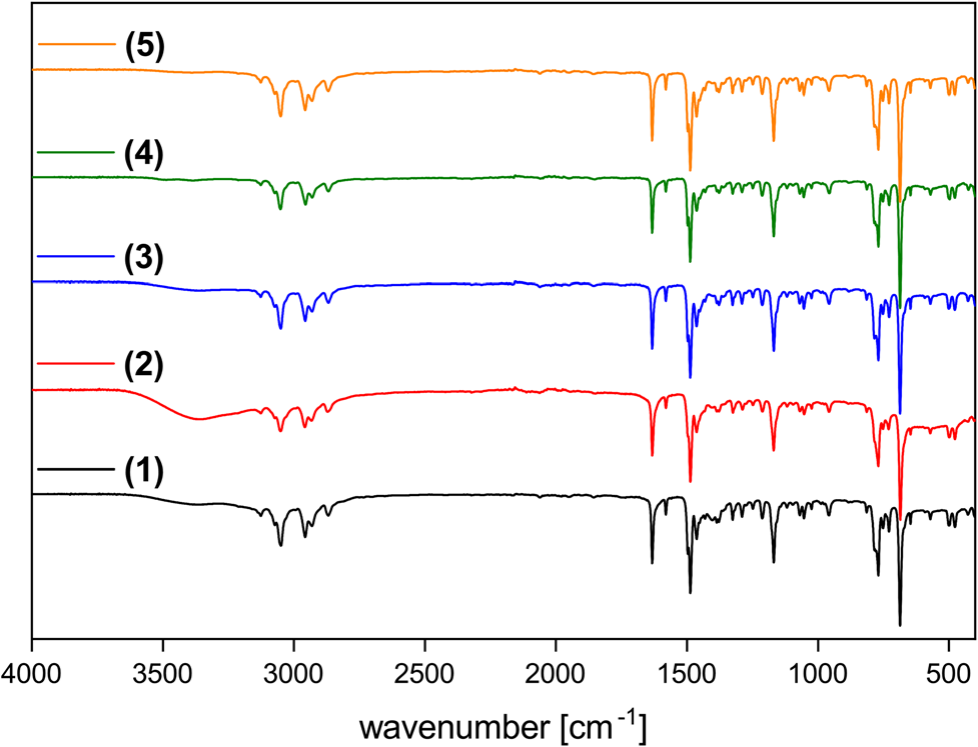
IR spectra of (C_4_Py)_2_[NiCl_4_] (1, black), (C_4_Py)_2_[Ni_0.5_Cu_0.5_Cl_4_] (2, black), (C_4_Py)_2_[Ni_0.5_Co_0.5_Cl_4_] (3, black), (C_4_Py)_2_[Ni_0.5_Mn_0.5_Cl_4_] (4, black) and (C_4_Py)_2_[Ni_0.5_Cu_0.5_Cl_4_] (5, black). Additional IR spectra of compounds 6, 7, 8 are shown in Fig. S20.† Data are shifted vertically to show all spectra clearly; *y*-axis shows transmission.

Fig. 5 shows representative Fourier-transform infrared (FT-IR) spectra of the ILs. Bands between 3100 and 2800 cm^−1^ can be assigned to vibrations in the *n*-butyl group and bands between 1650 and 1450 cm^−1^ can be assigned to vibrations of the pyridinium ring (aromatic C=N, C=C), in analogy to previous data.^28,33^ Additional broad bands between 3500 and 3100 cm^−1^ indicate the presence of water, thus confirming the ICP-OES data, Table 1.

**Fig. 6:**
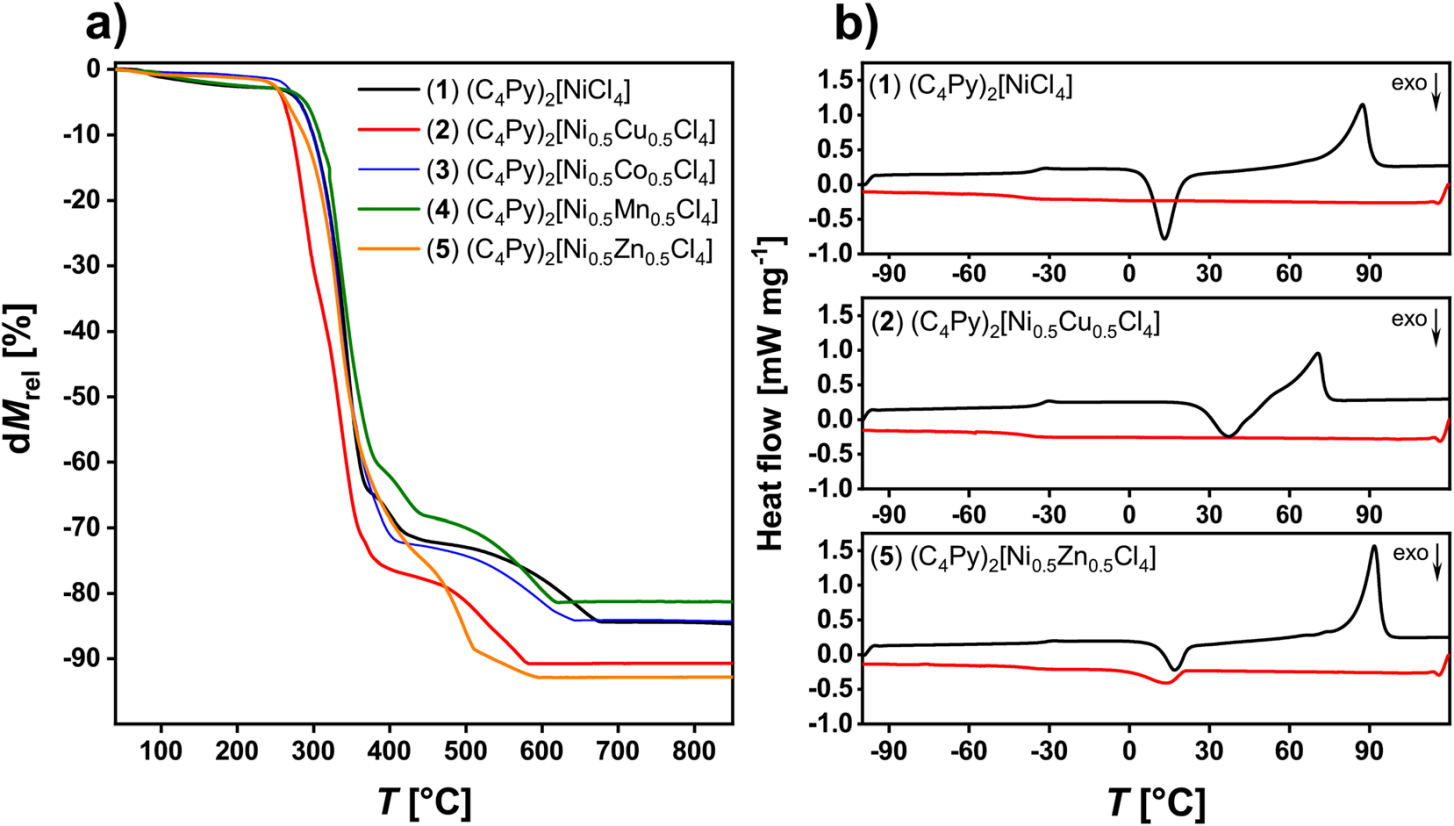
(a) TGA data of ILs 1 to 5 and (b) representative DSC data (2nd heating, black and 2nd cooling, red) of IL 1, 2, and 5.

The thermal stability of the ILs was analyzed *via* thermogravimetric analysis (TGA), Fig. 6a and S21.† Under conventional dynamic heating conditions with a heating rate of 10 K min^−1^, the ILs 1–8 are thermally stable up to *ca.* 250 °C. Most ILs show a first mass loss below 5% up to 200 °C. This loss can be attributed to water and solvent evaporation. Between 250 and *ca.* 370 °C a significant weight loss is observed. This loss can be attributed to the decomposition of the *N*-butylpyridinium ion, especially the elimination of the butyl side chain. The last two overlapping steps between *ca.* 370 and 800 °C can be attributed to further decomposition of the IL. Table 3 summarizes the TGA data for all compounds. Similar thermal properties were observed for imidazolium-based MILs including zinc and nickel. Here, Clarke *et al.*^34^ studied the thermal properties and stability of multiple MILs consisting of different metal halide anions and imidazolium or phosphonium-based cations. As shown here, the MILs presented by Clarke *et al.* are all thermally stable up to 200 °C. Moreover, a two-step degradation process shown in this publication is comparable to the one presented in the current article. This indicates a similar degradation mechanism including reverse Menshutkin decomposition, chloride dissociation and inorganic metal halide formation.

**Table 3:** DSC data of ILs 1–8^a^

IL	Heating						Cooling			
	*T*_g_^a^ [°C]	Δ*C*_p_ [J mol^−1^ K^−1^]	*T*_xt_^b^ [°C]	Δ*H* [kJ mol^−1^]	*T*_m_^c^ [°C]	Δ*H* [kJ mol^−1^]	*T*_g_^a^ [°C]	Δ*C*_p_ [J mol^−1^ K^−1^]	*T*_xt_^d^ [°C]	Δ*H* [kJ mol^−1^]
1	−31.9 ± 0.4	150.7 ± 12.6	6.6 ± 0.7	−24.1 ± 2.1	76.6 ± 0.4	29.0 ± 0.2	−60.2 ± 1.2	136.8 ± 11.0	−10.35	−3.6 ± 0.3
2	−35.5 ± 0.2	167.3 ± 2.3	26.4 ± 0.5	−18.8 ± 1.8	58.2 ± 0.3	18.1 ± 1.7	−44.4 ± 0.2	189.4 ± 3.8		
3					87.7 ± 0.3	31.6 ± 0.1			23.7 ± 5.0	−30.2 ± 0.1
4					94.1 ± 0.1	31.3 ± 0.3			46.0 ± 3.6	−30.2 ± 0.1
5	−33.4 ± 0.3	0.09 ± 0.02	9.5 ± 0.9	−19.1 ± 5.1	84.8 ± 0.2	31.3 ± 0.3	−55.4 ± 4.0	0.1 ± 0.0	−0.9 ± 3.0	−9.5 ± 6.8
6					79.5 ± 0.2	31.2 ± 0.1			33.4 ± 1.0	−30.0 ± 0.1
7					89.4 ± 0.4	31.1 ± 0.3			19.4 ± 2.3	−33.8 ± 0.3
8					94.8 ± 0.2	35.0 ± 0.2			40.5 ± 2.5	−34.4 ± 0.3

a All measurements were done with a heating rate of 10 K min^−1^ from −100 to 120 °C. All temperatures are the onset temperatures, [a] glass transition temperature, [b] melting temperature, [c] melting temperature (heating cycle), [d] crystallization temperature (cooling cycle). Empty fields indicate that transitions were not observed.

The phase behavior of the ILs 1–8 was determined *via* differential scanning calorimetry (DSC), Fig. 6b. Upon heating, some ILs exhibit glass transitions (*T*_g_) at around −35 °C, while some ILs do not exhibit any signal at these temperatures. Upon further heating, some ILs (ILs 1, 2 and 5) show a cold crystallization, typically at around 6 to *ca.* 26 °C. Again, this process is not observed for all compounds. Finally, all ILs exhibit a clear melting transition between 70 to 100 °C. Upon cooling, all ILs exhibit a strong undercooling typical of ILs. While most ILs do show a crystallization there is one example, IL 2, which does not show a direct crystallization signal. Also *T*_g_ is not always observed upon cooling. These observations are very similar to previous examples.^28,33^

**Fig. 7:**
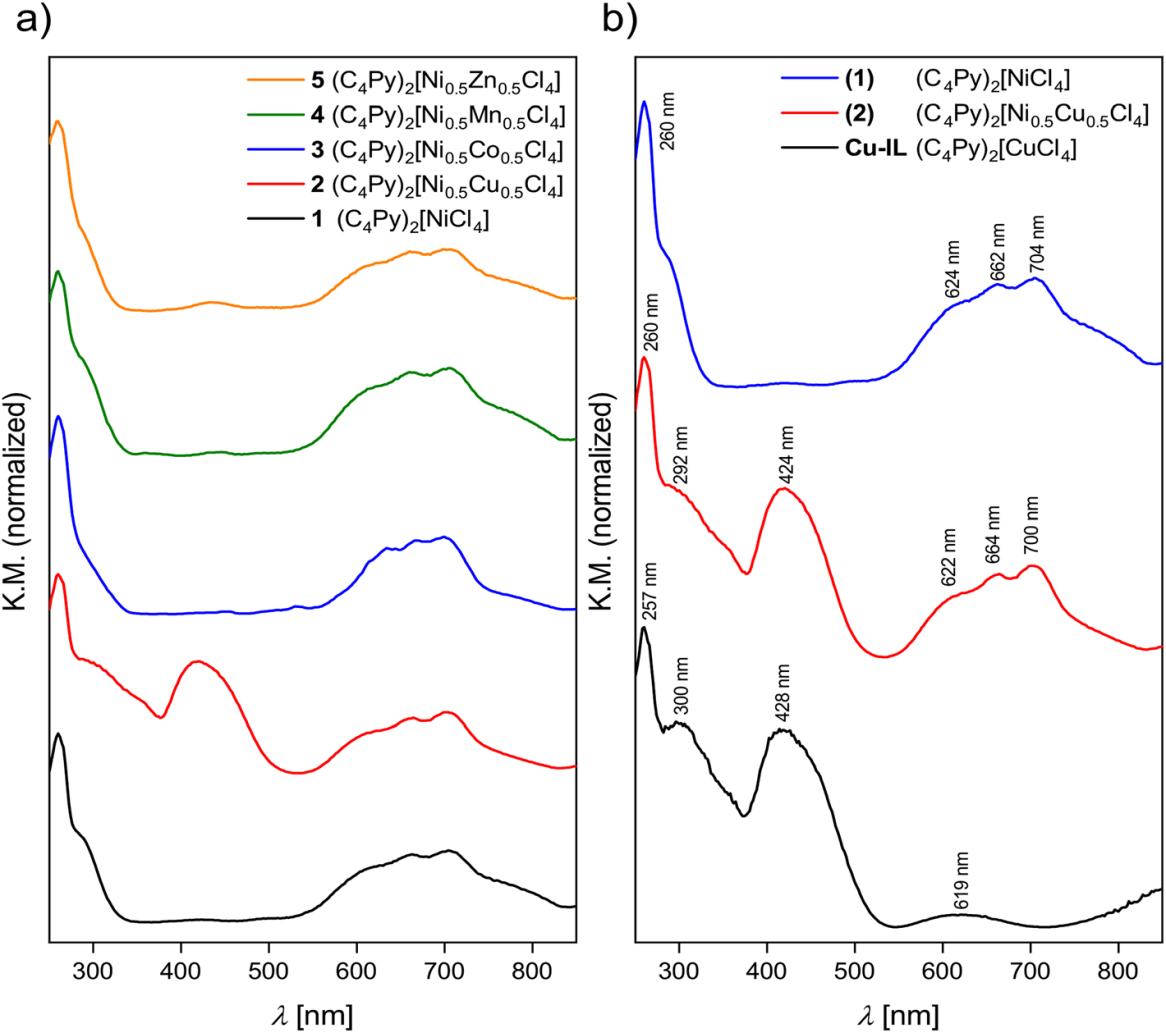
(a) UV-Vis reflectance spectra of the Ni-containing ILs 1–5, (b) comparison of the UV-Vis spectra of the two ILs based on Cu only and Ni only, (C_4_Py)_2_[CuCl_4_] and (C_4_Py)_2_[NiCl_4_], respectively, with the UV-Vis spectrum of the mixed compound (C_4_Py)_2_[Ni_0.5_Cu_0.5_Cl_4_], IL 2.

Fig. 7 shows UV-Vis solid-state reflectance measurements of the nickel-containing ILs. All ILs have an absorption band at *ca.* 260 nm, which stems from the n → π* and π → π* transitions of the cation.^28^ In addition to the typical bands from tetrahedrally coordinated Ni^2+^ between 550 and 750 nm, all spectra show additional bands that can be assigned to the second metal in the ILs. For example, in the case of IL 2, in addition to the transitions in the range from 550 to 750 nm that can be ascribed to the Ni^2+ 3^T_1_(F) → ^3^T_1_(P) transition,^35^ there is also a band at 424 nm. This transition can be assigned to the d–d transition of tetrahedrally coordinated Cu^2+^.^36–38^

In the case of IL 3 the absorption bands of the Co^2+^ and Ni^2+^ transitions overlap between 550 to 750 nm and cannot be observed separately. In the case of the Ni^2+^/Mn^2+^ combination, essentially only the Ni-centered transitions can be observed and the Mn-centered signals are very weak. This is due to the well-known fact that d–d transitions in Mn^2+^ are parity forbidden.^39^ Zn^2+^ does not contribute to the spectra because these are d^10^ systems and therefore no transitions can be observed in the respective spectra. The combination of the two contributions to the overall spectra is demonstrated in Fig. 7b for the Ni/Cu pair. Spectra of all ILs are shown in Fig. S22.†

**Fig. 8:**
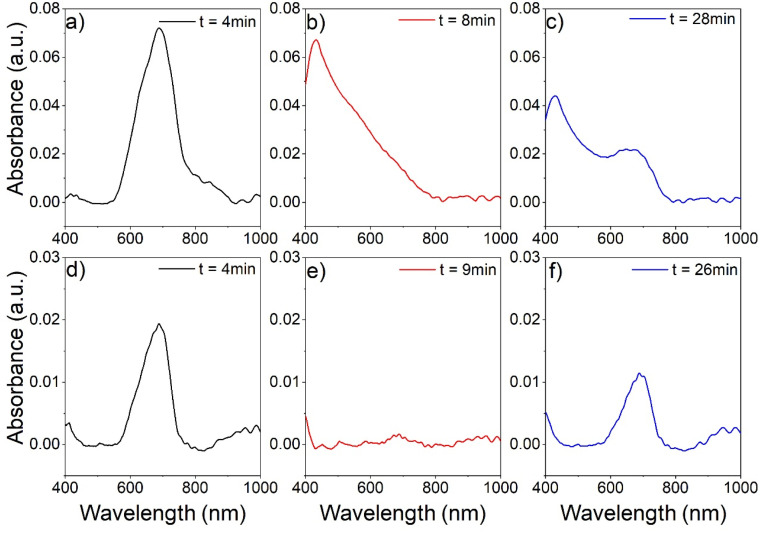
Analysis of the absorbance spectra of thin layers of (C_4_Py)_2_[Ni_0.5_Zn_0.5_Cl_4_] on glass used as gas sensors which were exposed in sequence to different gas mixtures: first, the gas sensors were exposed to a pure stream of dry N_2_ (0–5 min, (a and d)). Afterwards, ammonia vapor (b) or water vapor (wet N_2_, (e)) was mixed to the nitrogen stream (5–10 min). Finally, the gas sensors were again exposed to a stream of pure dry N_2_ (c and f).

Since the central unit in these ILs is essentially a metal complex, ligand exchange reactions, *e.g.*, with gaseous molecules, can occur. These ligand exchange or addition reactions can be accompanied by quite strong color changes, and application in gas sensing would be possible.^40^ A previous study by Bagdahn *et al.* of a copper-based MIL-hybrid material showed a color change after the material was exposed to ammonia vapor.^41^ For example, Fig. 8 shows the analysis of the absorbance of a layer of (C_4_Py)_2_[Ni_0.5_Zn_0.5_Cl_4_] deposited on glass and exposed to nitrogen-assisted ammonia vapor. The sample was placed in a transparent flow cell and then exposed to a stream of N_2_ for 5 minutes. Fig. 8a shows the absorption spectrum of the sample in the N_2_ stream (4 minutes after the start of the experiment). Only one main peak is observed at 680 nm. After three minutes of exposure to ammonia vapor (minute 8 of the experiment, Fig. 8b), the peak at 680 nm has disappeared and an intense peak at 420 nm predominates in the absorption spectrum. In minute 10 of the experiment, the gas stream is switched back to pure N_2_. Fig. 8c shows the absorption spectrum of the sample after 28 minutes of the experiment, corresponding to the ammonia desorption phase. This spectrum is a convolution of the two previously observed peaks, with the peak at 420 nm losing intensity and the peak at 680 nm gaining intensity.

However, examination of the change in the absorption spectrum as a function of time shows that the spectrum in Fig. 8c has reached a nearly steady state. A similar analysis was performed with wet N_2_ (nitrogen stream containing water vapor). As in the previous case, the ionic liquid layer shows a main absorption peak at 680 nm at the beginning of the analysis (Fig. 8d). When the sample is exposed to wet N_2_ (Fig. 8e), the intensity of the peak decreases. However, the appearance of another peak at 420 nm as in the case of ammonia exposure is not observed. In the desorption phase (Fig. 8f), the peak at 680 nm recovers by slightly more than 50% of the original intensity. A more comprehensive analysis of the behavior of the absorption peaks in the presence of ammonia or wet nitrogen is presented in the ESI Fig. S23,† as well as the behavior of other ionic liquids.

## Discussion

We have previously shown that metal containing ILs can be modified by combining more than one metal into the MX_4_^2−^ anion, even at different ratios. For example, proper combination of metals enables the synthesis of ILs exhibiting colors from light blue to red.^28^ The current article explores two further aspects: (1) the use of additional d-metals that have not been studied in detail so far and (2) the exploitation of the UV-Vis absorption of these ILs to generate a prototype optical sensor for ammonia gas sensing.

Clearly, the addition of further metal ions to the existing pool of M^2+^ (M = Cu, Co, Zn, *etc.*) broadens the accessible color range and the current ILs exhibit additional colors, especially in the blue to green range, Fig. 1. This simple observation can also be corroborated by solid state UV-Vis spectroscopy. The spectra of the ILs are a combination of the spectra of the individual d-metal present in the ILs, Fig. 7 and S22.† As a result, the combination of two different metal ions in the current ILs provides an ever increasing pool of strongly colored ILs with melting points below *ca.* 100 °C, Fig. 6, Table 3. Finally, ICP OES (Table 1) and IR spectroscopy (Fig. 5, S20†) show that while some ILs take up some water, the uptake is reversible and all solvent or water is released below 200 °C, consistent with TGA, Fig. 6a and S21.†

At this point it must be noted that, contrary to previous data,^28^ these ILs do not exhibit a band gap. Rather, the UV-Vis spectra clearly show that there are individual contributions from separated metal centers and there is no indication of the formation of bands or band gaps.

The crystal structures are closely related to the existing examples^23,28,33,42^ and these data clearly show that the process and approach towards these ILs is highly reproducible, flexible, and adaptable. Moreover, like on the previous examples, all structures of the single crystals (Fig. 2, 3, Table 1, Fig. S1–S18, Tables S1–S3†) match those observed in the powders isolated directly from the synthesis (Fig. 4 and S19†).

As a result, the approach towards multi-metallic ILs is extremely flexible, adaptable, and robust. Moreover, the optical properties lend themselves for exploitation in, *e.g.*, sensing or gas sequestration. Indeed, our preliminary experiments (Fig. 8 and S23†) show that an efficient gas detection can be realized using a very simple optical setup. This clearly demonstrates the high potential of multi-metallic ILs for the development of, *e.g.* gas sensors. However, a detailed analysis of the data shows that there is a need to further develop and optimize the gas sensor design. For example, the variation in optical response of different ionic liquids to ammonia vapor and water vapor suggest (see Fig. S23 and S24†) to explore the potential of arrays of colorimetric sensors based on different ionic liquids for detecting volatile gases and their mixtures in air (optical nose).^43^

Besides sensing, one must not forget that (ammonia) gas sequestration or gas separation (membranes) are topics of high current interest.^44,45^ Given the fact that the ammonia uptake is quite effective, these materials could also be candidates for gas sequestration, but this is a subject beyond the scope of this article and will be evaluated in the future.

## Conclusion

The current study further expands the pool of metal-based ILs, in particular in terms of the color and chemical space. The process towards these interesting and diverse ILs is extremely robust and highly flexible. The ILs can easily be adapted to a certain property and composition and the synthesis can easily be scaled to (at least) a multi-gram scale. Moreover, the ILs can be processed into a sensor suitable for the detection of ammonia (other gases will certainly follow suit) and the system therefore represents a versatile prototype for a highly flexible and adaptable sensor platform or possibly for gas sequestration.

## Experimental section

### Materials

*N*-Butylpyridinium chloride (≥98%, Merck, CAS), CuCl_2_ (99.995%, ABCR, CAS), CoCl_2_ (99.95%, ABCR, CAS), MnCl_2_ (99.95%, ABCR, CAS), NiCl_2_ (99.95%, ABCR, CAS), ZnCl_2_ (99.95%, ABCR, CAS), 2-propanol (99.5%, Carl Roth, CAS) and hydrochloric acid (37%, VWR Chemicals, CAS) were used without further purification.

### General IL synthesis route

IL synthesis was done according to ref. 25. The synthesis procedure was repeated up to five times for reproducibility. 2.00 g (8.31 mmol) of *N*-butylpyridinium chloride were dissolved in 50 mL 2-propanol. For monometallic and bimetallic ILs, 4.16 mmol of anhydrous metal salts (M = Cu, Co, Mn, Ni, Zn) and 0.1 mL of HCl (37%) was added to the solution and the mixture was stirred for 30 to 60 minutes under reflux. The solvent was removed by rotary evaporation and the resulting products were dried under vacuum (10^−3^ mbar). The products were used without further purification. For single crystal growth, a few grains of the compounds were dissolved in various solvents, *e.g.* methanol, acetonitrile, or 2-propanol. The solvents were allowed to slowly evaporate and after several days to weeks, crystals could be collected.

#### (C_4_Py)_2_[NiCl_4_] (1)

Yield: 2.62 g (95%); M [g mol^−1^] 472.94; ICP OES (in %): Ni 11.53, found: Ni 11.23 ± 0.15; FT-IR (ATR, cm^−1^): 3127, 3051, 2960, 2928, 2868, 1631, 1579, 1488, 1461, 1385, 1328, 1290, 1258, 1213, 1168, 1010, 789, 729, 686, 499, 478; solid state UV-Vis (nm): 260, 422, 662, 704.

#### (C_4_Py)_2_[Ni_0.5_Cu_0.5_Cl_4_] (2)

Yield: 2.58 g (93%); M [g mol^−1^] 475.36; ICP OES (in %): Ni 6.17, Co 6.68, found: Cu 6.16 ± 0.65, Ni 5.68 ± 0.58; FT-IR (ATR, cm^−1^): 3128, 3054, 2961, 2931, 2873, 1633, 1581, 1489, 1456, 1388, 1325, 1289, 1249, 1212, 1171, 1070, 1054, 959, 813, 772, 732, 688, 499, 478; solid state UV-Vis (nm): 260, 420, 664, 700.

#### (C_4_Py)_2_[Ni_0.5_Co_0.5_Cl_4_] (3)

Yield: 2.51 g (91%); M [g mol^−1^] 473.06; ICP OES (in %): Ni 6.20, Co 6.23, found: Ni 6.69 ± 0.56, Co 5.77 ± 0.68; FT-IR (ATR, cm^−1^): 3127, 3050, 2958, 2931, 2869, 1632, 1580, 1487, 1464, 1377, 1326, 1290, 1248, 1211, 1168, 1069, 1054, 958, 814, 770, 729, 688, 499, 476; solid state UV-Vis (nm): 260, 450, 530, 634, 670, 700.

#### (C_4_Py)_2_[Ni_0.5_Mn_0.5_Cl_4_] (4)

Yield: 2.58 g (94%); M [g mol^−1^] 471.06; ICP OES (in %): Ni 6.23, Co 5.83, found: Ni 6.54 ± 0.28, Mn 5.62 ± 0.14; FT-IR (ATR, cm^−1^): 3129, 3054, 2959, 2933, 2873, 1633, 1581, 1488, 1464, 1387, 1326, 1290, 1249, 1213, 1170, 1070, 1054, 957, 814, 770, 729, 688, 498, 473; solid state UV-Vis (nm): 260, 360, 446, 660, 706.

#### (C_4_Py)_2_[Ni_0.5_Zn_0.5_Cl_4_] (5)

Yield: 2.64 g (95%); M [g mol^−1^] 476.28; ICP OES (in %): Ni 6.16, Zn 6.86, found: Ni 5.99 ± 0.38, Zn 6.97 ± 0.33; FT-IR (ATR, cm^−1^): 3127, 3051, 2958, 2932, 2872, 1632, 1487, 1463, 1386, 1325, 1288, 1248, 1212, 1168, 1067, 1056, 1025, 953, 812 769, 729, 687; solid state UV-Vis (nm): 260, 432, 488, 505, 660, 706.

#### (C_4_Py)_2_[Zn_0.5_Cu_0.5_Cl_4_] (6)

Yield: 2.57 g (92%); M [g mol^−1^] 478.21; ICP OES (in %): Zn 6.83, Cu 6.64, found: Cu 6.57 ± 0.13, Zn 6.31 ± 0.48; FT-IR (ATR, cm^−1^): 3128, 3055, 2959, 2933, 2872, 1633, 1581, 1488, 1464, 1379, 1324, 1289, 1247, 1212, 1170, 1071, 1053, 958, 614, 772, 731, 687, 499, 478; solid state UV-Vis (nm): 260, 296, 322, 422.

#### (C_4_Py)_2_[Zn_0.5_Co_0.5_Cl_4_] (7)

Yield: 2.64 g (95%); M [g mol^−1^] 476.40; ICP OES (in %): Zn 6.86, Co 6.19, found: Zn 6.85 ± 0.28, Co 6.18 ± 0.34; FT-IR (ATR, cm^−1^): 3129, 3054, 2959, 2933, 2871, 1633, 1582, 1488, 1464, 1378, 1325, 1290, 1249, 1213, 1169, 1064, 1053, 958, 814, 772, 729, 687, 499, 476; solid state UV-Vis (nm): 262, 366, 408, 452, 530, 634, 670, 700.

#### (C_4_Py)_2_[Zn_0.5_Mn_0.5_Cl_4_] (8)

Yield: 2.38 g (86%); M [g mol^−1^] 474.40; ICP OES (in %): Zn 6.89, Mn 5.79, found: Zn 6.82 ± 0.07, Mn 5.58 ± 0.05; FT-IR (ATR, cm^−1^): 3128, 3052, 2958, 2932, 2871, 1632, 1580, 1487, 1463, 1377, 1325, 1290, 1260, 1212, 1168, 1094, 1069, 1054, 957, 769, 729, 687, 497, 478; solid state UV-Vis (nm): 260, 358, 432, 444.

### Inductively coupled plasma optical emission spectrometry

ICP OES measurements were performed on a PerkinElmer Optical Emission Spectrometer Optima 5300 DV (Scott-Chamber/Cross-Flow-Nebulizer). Read time was 2 to 10 s with a power of 1400 W and plasma gas flow of 17 L min^−1^, auxiliary gas flow of 0.2 L min^−1^ and nebulizer gas flow of 0.6 L min^−1^. The measurement was performed axially. The signals of Cu, Co, Mn, Ni and Zn were observed at *λ* = 327.393, 238.892, 257.610, 231.604, and 213.8 nm. The metal content of the ILs was measured three times per sample and two different batches of the ILs were used for comparability.

### Single-crystal X-ray diffraction

Suitable single crystals were selected using a light microscope and separated with perfluoropolyalkylether oil. The X-ray diffraction experiment was carried out on a Stoe Stadivari (four circle goniometer) with a Genix Microfocus X-ray source (Mo-Kα-radiation, *λ* = 0.71073 Å) and a Pilatus 200 K detector. The measurements were done at 210 K using an Oxford Cryosteam cooling device. The data were corrected for absorption as well as for Lorentz and polarization effects using the program *X*-area and the structure was solved by direct methods and refined against *F*^2^ on all data by full-matrix least-squares using the SHELX suite of programs.^46,47^ The crystal structure was visualized with Diamond.^48^ The crystallographic data (1: CCDC 2157870; 2: CCDC 2157871; 3: CCDC 2157879) are available from the CCDC website.

### Powder X-ray diffraction

XRD data were collected on a Panalytical Empyrean powder X-ray diffractometer operating at 40 kV and 40 mA. The diffractometer was configured with a focusing X-ray mirror for Cu radiation (*λ* = 1.5419 Å) and a pixcel 1D detector. Scans were performed from 4 to 70° in 2*θ* with a step size of 0.0131°.

### Infrared spectroscopy

IR spectra were recorded on a Thermo Nicolet FT-IR Nexus with a SmartOrbit ATR attachment from 4000 to 400 cm^−1^ with a resolution of 4 cm^−1^ and 64 scans. The resulting data were evaluated and refined (H_2_O, CO_2_ and ATR correction) with the program Omnic V6.2 (Thermo Nicolet).

### Thermogravimetric analysis

TGA was done on a PerkinElmer TGA 4000 in air from room temperature to 900 °C. The heating rate was 10 K min^−1^ and data analysis was done with the Pyris software package (PerkinElmer).

### Differential scanning calorimetry

DSC measurements were performed on a Neztsch DSC 214 Polyma with heating rates of 1, 2, 5, or 10 K min^−1^ under nitrogen. A total of four cycles were measured for each sample. The resulting data were evaluated with the Proteus V7.1.0 software (Netzsch).

### UV-Vis spectroscopy and sensor prototype

A 0.21 mol L^−1^ solution of the IL (C_4_Py)_2_[Ni_0.5_Zn_0.5_Cl_4_] in methanol was prepared. Microscopy glass slides (20 × 20 mm^2^, Carl Roth) were treated with piranha solution (mixture of 96% H_2_SO_4_ and 30% H_2_O_2_, ratio 3 : 1, v/v) for at least 2 hours, cleaned with deionized water, and dried in a stream of nitrogen. Afterwards, 80 μL of the methanolic solution were deposited on the glass slides and distributed as uniformly as possible over the complete surface of the slide with a pressure modulated stream of air.

Improved distribution of the IL on the glass slide was achieved by spin-coating using a custom-made spin coater with a DC Power Supply (Voltcraft, Model 2256) for 5 minutes with 13 V, which refers to ∼310 rpm. The slides were left to dry upright to avoid the formation of drying traces.

Before the measurement the coated glass slides were pretreated in the drying oven at 50 °C for several minutes and then directly inserted into a flow cell. They were flushed with nitrogen for 5 minutes. After that they were flushed for another 5 minutes with nitrogen that was bubbled through 5 mL of aqueous ammonia solution (25%, AnalaR NORMAPUR, VWR Chemicals). Afterwards, the glass slides were again flushed with nitrogen for 20 minutes. As a control experiment, the same measurements were also performed with 5 mL of water (here called wet nitrogen) instead of aqueous ammonia solution. The experimental setup can be found in Fig. S25.† All sensing experiments were repeated 3 times showing a high level of repeatability.

Time-resolved UV-Vis spectra were then recorded for 5 minutes with an absorbance spectrum collected every 2 seconds. UV/Vis spectra were measured using an Ocean Optics QE65 Pro Spectrometer (Ostfildern, Germany) with an Ocean Optics Halogen Lightsource HL-2000 (Ostfildern, Germany). The light source contained two filters (Absorptive ND Filter, NE21OB and NE205B, Thorlabs GmbH, Bergkirchen, Germany) to decrease the light intensity. The distance between the optical fiber coming from the light source and the sample was 12 cm and the distance between the sample and the optical fiber going to the spectrometer was 8 cm. The measurements were carried out using the program Ocean View (integration time 8 ms, scans to average 25, boxcar width 5, trigger mode: on demand, electric dark correction enabled, non-linearity correction disabled) and evaluated with the program Igor Pro 9.00 using the macro provided by Sailor.^49,50^

## Conflicts of interest

There are no conflicts to declare.

## Supplementary Material

RA-012-D2RA05581C-s001

RA-012-D2RA05581C-s002
