# Supplementary material for: Mixed chloridometallate(ii) ionic liquids with tunable color and optical response for potential ammonia sensors
Source: RSC Adv. 2022 Dec 8;12(54):35072–82. doi: 10.1039/d2ra05581c (PMC9729802; doi:10.1039/d2ra05581c)
Supplement: RA-012-D2RA05581C-s001 [file RA-012-D2RA05581C-s001.pdf]

Electronic Supplementary Material (ESI) for RSC Advances.
This journal is © The Royal Society of Chemistry 2022

Supporting Information

New mixed halidometallate ionic liquids with tuneable color and optical response for ammonia sensing and sequestration

Karsten Behrens,[a] Christian Balischewski,[a] Eric Sperlich,[a] Antonia Menski,[a] Ruth Fabiola Balderas-Valadez,[a] Claudia Pacholski,[a] Christina Günter,[b] Susanne Lubahn,[a] Alexandra Kelling,[a] and Andreas Taubert*[a]

[a] Institute of Chemistry, University of Potsdam, Karl-Liebknecht-Strasse 24-25, D-14476 Potsdam, Germany.

[b] Institute of Geosciences, University of Potsdam, Karl-Liebknecht-Strasse 24-25, D-14476 Potsdam, Germany.

In this section, further images and information of the crystal structures of the compounds $(C_4Py)_2[NiCl_4]$ **1**, $(C_4Py)_2[Ni_{0.5}Co_{0.5}Cl_4]$ **3** and $(C_4Py)_2[Zn_{0.54}Co_{0.46}Cl_4]$ **7** are shown. The first image of each compound (**1**: Figure 1; **3**: Figure 7; **7**: Figure 13) shows the molecule in ellipsoid view with atomic labels. This is followed by pictures showing the hydrogen bonds that occur (**1**: Figure 2; **3**: Figure 8; **7**: Figure 14), tables with the geometrical data of the hydrogen bonds (**1**: Table 1; **3**: Table 2; **7**: Table 3) and the anion-π interactions in the compounds (**1**: Figure 3; **3**: Figure 9; **7**: Figure 15). In addition, the cell view is shown for each compound (**1**: Figure 4, Figure 5 and Figure 6; **3**: Figure 10, Figure 11 and Figure 12; **7**: Figure 16, Figure 17 and Figure 18).

In compound **3**, the occupation of the transition metal of the anion was fixed at 50% Ni and 50% Co, because free refinement was not possible. The 50/50 ratio was verified by ICP-OES analysis. In compounds **7** only the positions and the anisotropic displacement parameters (ADP) of the transition metals were freely refined, i.e. the Zn and Co atoms were split, but not the chloride atoms. This resulted in a Zn/Co ratio of 54/46 for compound **7**. Due to the similar positions of the transition metal atoms and the nearly equal number of electrons, refinement of the composition is very uncertain. In addition, the ratio of the two transition metals also varies in different crystals. For this reason, the ICP-OES analysis results are more accurate with regard to the ratio of the two transition metal types in the compounds.

All three structures of the compounds are isotypic to each other, the arrangement of the cations and anions is similar in the solid state. This results in the formation of weak hydrogen bonds between the protons of the cations and the chloride ligands of the anions. In the corresponding pictures (**1**: Figure 2; **3**: Figure 8; **7**: Figure 14) all hydrogen bonds up to a proton-acceptor distance of 2.8 Å are shown. For compound **1** there are five, for **3** six and for **7** five such hydrogen bonds. These C-H···Cl hydrogen bonds are similar in strength to those reported in the literature.[1–3] Furthermore, so-called anion-π interactions are formed between the aromatic rings of the cations and the chloride ligands of the anions (**1**: Figure 3; **3**: Figure 9; **7**: Figure 15;). The chloride ligands are oriented at a distance of 4.0 to 4.3 Å above the center of the aromatic ring. Each of the eight symmetry independent chloride ligand forms such an anion-π interaction and all four symmetry-independent aromatic centers *X1-X4* are involved in two such interactions, resulting in intermolecular chains. Such interactions are very often observed between N-heterocyclic compounds and anions and the values correspond to those given in the literature.[4–6]

Compound 1:

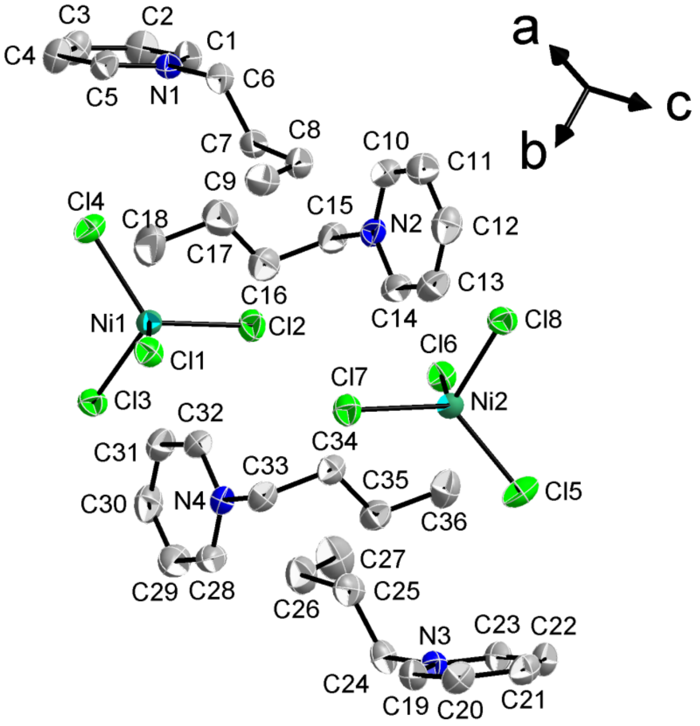

Figure S1: Structure of **1** with atomic labels, hydrogen atoms were omitted. Displacement ellipsoids are shown at the 50% probability level.

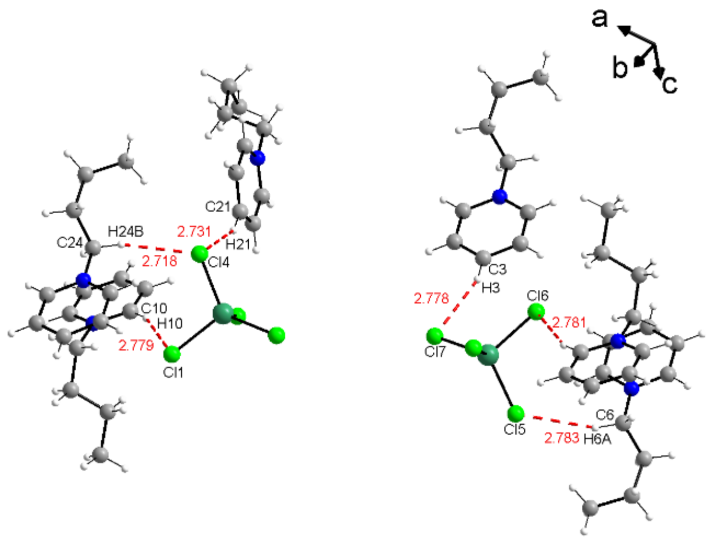

Figure S2: Hydrogen bonds between the cations and the anions in **1**. Only hydrogen bonds with a hydrogen-acceptor distance up to 2.8 Å are shown.

Table S1: Geometrical data for the intermolecular hydrogen bonds of $(C_4Py)_2[NiCl_4]$ **1**.

D—H···*A*	*D*—H	H···*A*	*D*···*A*	*D*—H···*A*
C1—H1···Cl1^{i}	0.94	2.88	3.5623 (19)	131
C1—H1···Cl3^{i}	0.94	2.81	3.5989 (19)	142
C4—H4···Cl6^{ii}	0.94	2.91	3.584 (2)	130
C5—H5···Cl5^{iii}	0.94	2.96	3.7762 (19)	147
C6—H6*A*···Cl5^{iii}	0.98	2.78	3.7187 (19)	160
C6—H6*B*···Cl3^{i}	0.98	2.84	3.6100 (19)	136
C7—H7*B*···Cl4	0.98	2.88	3.7631 (19)	150
C10—H10···Cl1^{i}	0.94	2.78	3.678 (2)	161
C14—H14···Cl7	0.94	2.91	3.767 (2)	152
C15—H15*A*···Cl7	0.98	2.91	3.602 (2)	128
C15—H15*B*···Cl1^{i}	0.98	2.95	3.837 (2)	152
C19—H19···Cl6^{iv}	0.94	2.88	3.5755 (19)	132
C19—H19···Cl8^{iv}	0.94	2.87	3.7039 (19)	148
C22—H22···Cl3^{v}	0.94	2.93	3.5838 (19)	128
C23—H23···Cl4^{vi}	0.94	2.93	3.7504 (19)	147
C24—H24*A*···Cl8^{iv}	0.98	2.82	3.6106 (19)	139
C24—H24*B*···Cl4^{vi}	0.98	2.72	3.6732 (19)	165
C28—H28···Cl6^{iv}	0.94	2.78	3.685 (2)	162
C29—H29···Cl8^{iv}	0.94	2.98	3.639 (2)	129
C31—H31···Cl1^{vii}	0.94	2.90	3.590 (2)	131
C32—H32···Cl2	0.94	2.94	3.796 (2)	152
C33—H33*A*···Cl6^{iv}	0.98	2.97	3.8632 (19)	152
C33—H33*B*···Cl2	0.98	2.92	3.606 (2)	128

Symmetry codes: (i) −*x*+3/2, *y*−1/2, −*z*+3/2; (ii) −*x*+1, −*y*, −*z*+1; (iii) *x*+1, *y*, *z*; (iv) −*x*+1/2, *y*+1/2, −*z*+3/2; (v) *x*−1/2, −*y*+1/2, *z*+1/2; (vi) *x*−1, *y*, *z*; (vii) *x*−1/2, −*y*+1/2, *z*−1/2.

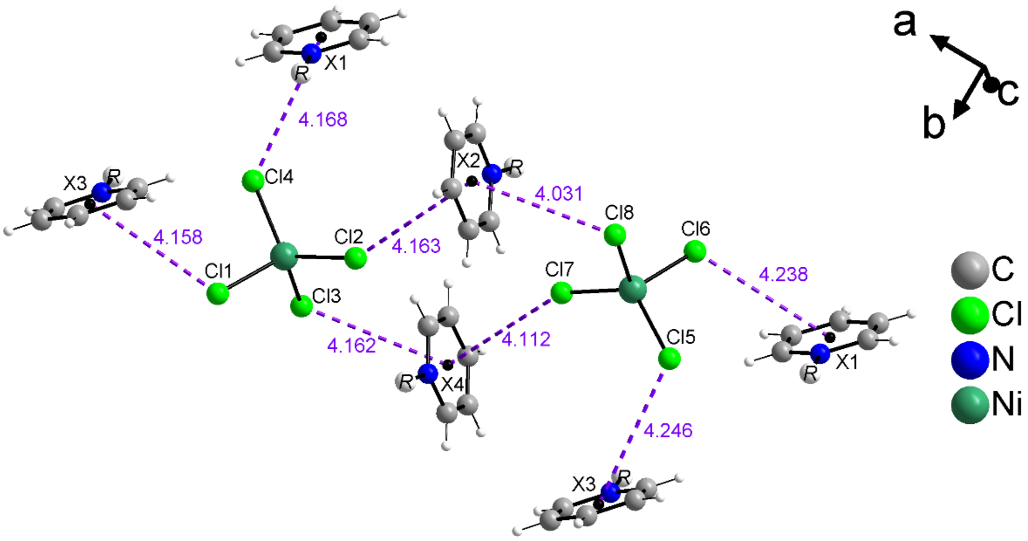

Figure S3: Anion-π interactions between pyridine rings of the cations and chloride ligands of the anions in **1**. Different aromatic centers are labeled as X1 to X4, the butyl substituents of the cations are omitted and shown as R.

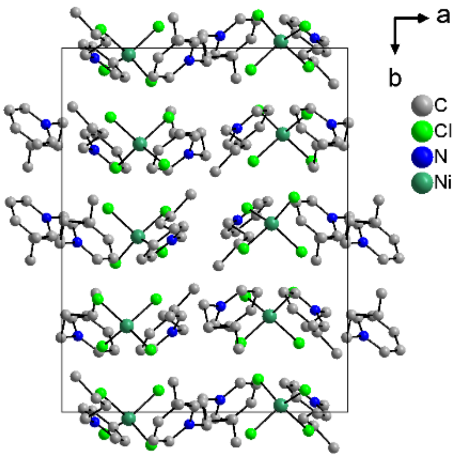

Figure S4: Packing of the compound **1** with view along the c axis (hydrogen atoms were omitted).

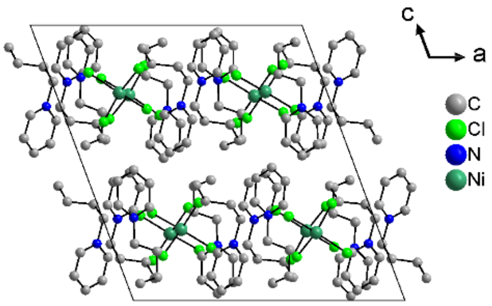

Figure S5: Packing of the compound **1** with view along the b axis (hydrogen atoms were omitted).

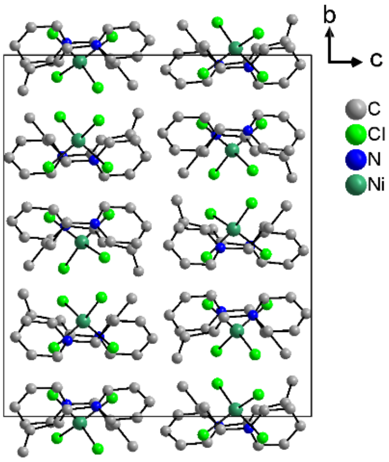

Figure S6: Packing of the compound **1** with view along the a axis (hydrogen atoms were omitted).

Compound 3:

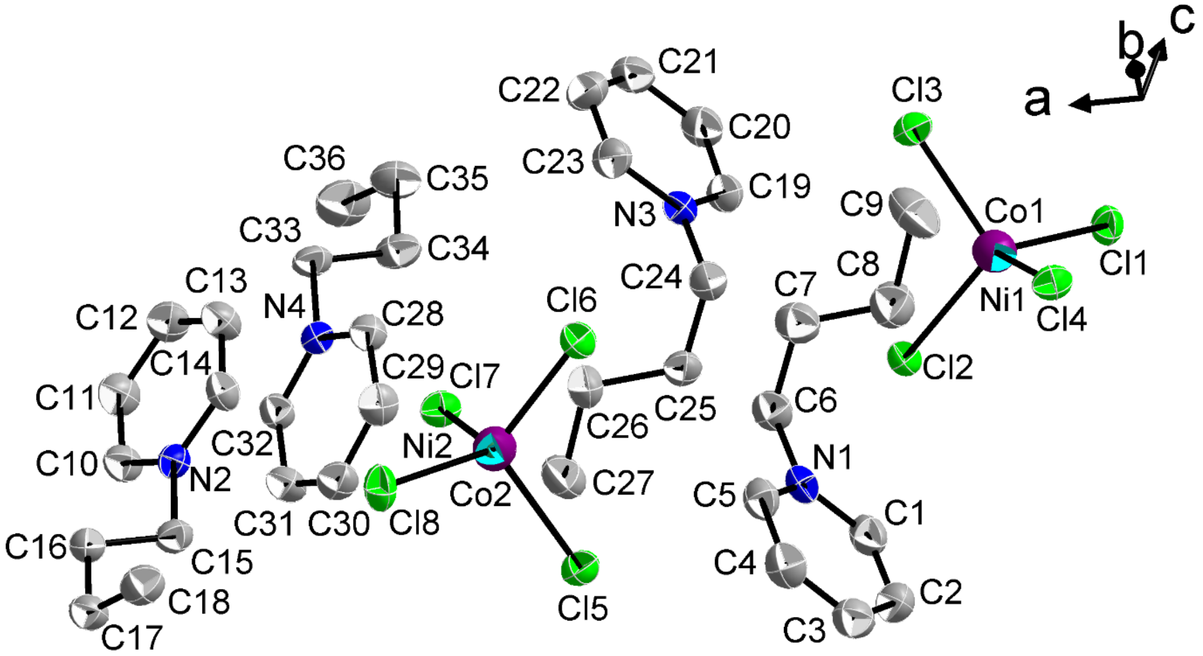

Figure S7: Structure of **3** with atomic labels, the mixed occupation of Ni and Co is shown, hydrogen atoms were omitted. Displacement ellipsoids are shown at the 50% probability level.

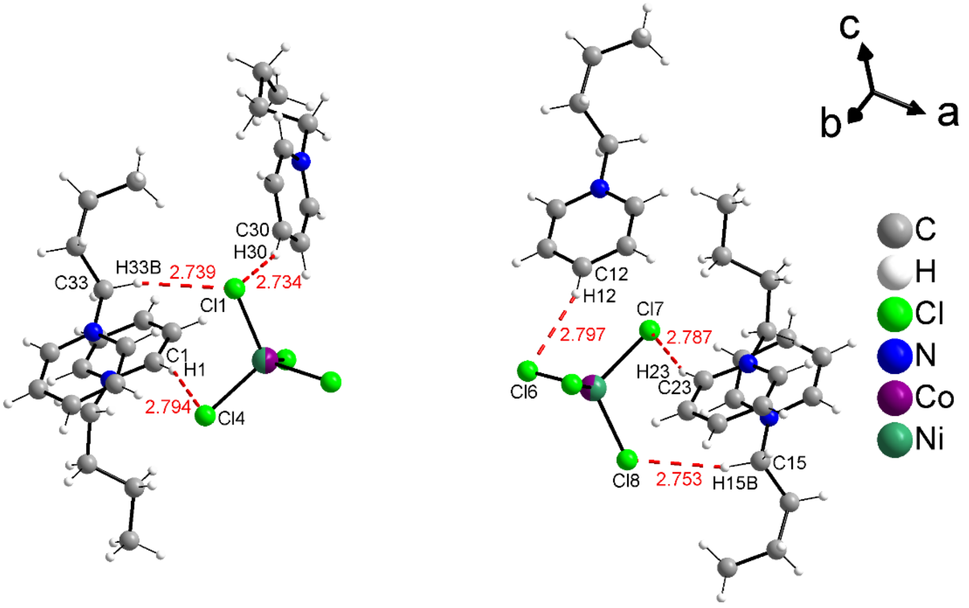

Figure S8: Hydrogen bonds between the cations and the anions in **3**. Only hydrogen bonds with a hydrogen-acceptor distance up to 2.8 Å are shown.

Table S2: Geometrical data for the intermolecular hydrogen bonds of $(C_4Py)_2[Ni_{0.5}Co_{0.5}Cl_4]$ **3**.

D—H···*A*	*D*—H	H···*A*	*D*···*A*	*D*—H···*A*
C1—H1···Cl4^{i}	0.94	2.79	3.696 (2)	161
C5—H5···Cl6	0.94	2.89	3.750 (2)	153
C6—H6*A*···Cl6	0.98	2.92	3.614 (2)	129
C10—H10···Cl4^{ii}	0.94	2.91	3.586 (2)	130
C13—H13···Cl7^{iii}	0.94	2.88	3.573 (2)	131
C14—H14···Cl8	0.94	2.97	3.797 (2)	147
C15—H15*A*···Cl3^{ii}	1.00 (2)	2.86 (2)	3.632 (2)	134.4 (15)
C15—H15*B*···Cl8	1.02 (2)	2.75 (2)	3.743 (2)	165.0 (16)
C16—H16*B*···Cl1^{iv}	0.98	2.86	3.738 (2)	150
C19—H19···Cl2	0.94	2.93	3.790 (2)	153
C20—H20···Cl4^{v}	0.94	2.88	3.570 (2)	131
C22—H22···Cl5^{vi}	0.94	2.98	3.649 (3)	130
C23—H23···Cl7^{vi}	0.94	2.79	3.691 (2)	162
C24—H24*A*···Cl7^{vi}	0.98	2.98	3.868 (2)	152
C24—H24*B*···Cl2	0.98	2.92	3.615 (2)	129
C28—H28···Cl5^{vi}	0.94	2.87	3.710 (2)	149
C28—H28···Cl7^{vi}	0.94	2.90	3.587 (2)	131
C31—H31···Cl3^{vii}	0.94	2.90	3.564 (2)	129
C32—H32···Cl1^{iv}	0.94	2.93	3.760 (2)	147
C33—H33*A*···Cl5^{vi}	0.98	2.84	3.627 (2)	138
C33—H33*B*···Cl1^{iv}	0.98	2.74	3.693 (2)	165

Symmetry codes: (i) −x+1/2, y−1/2, −z+3/2; (ii) −x+3/2, y−1/2, −z+3/2; (iii) −x+2, −y, −z+2; (iv) x+1, y, z; (v) x+1/2, −y+1/2, z+1/2; (vi) −x+3/2, y+1/2, −z+3/2; (vii) x+1/2, −y+1/2, z−1/2.

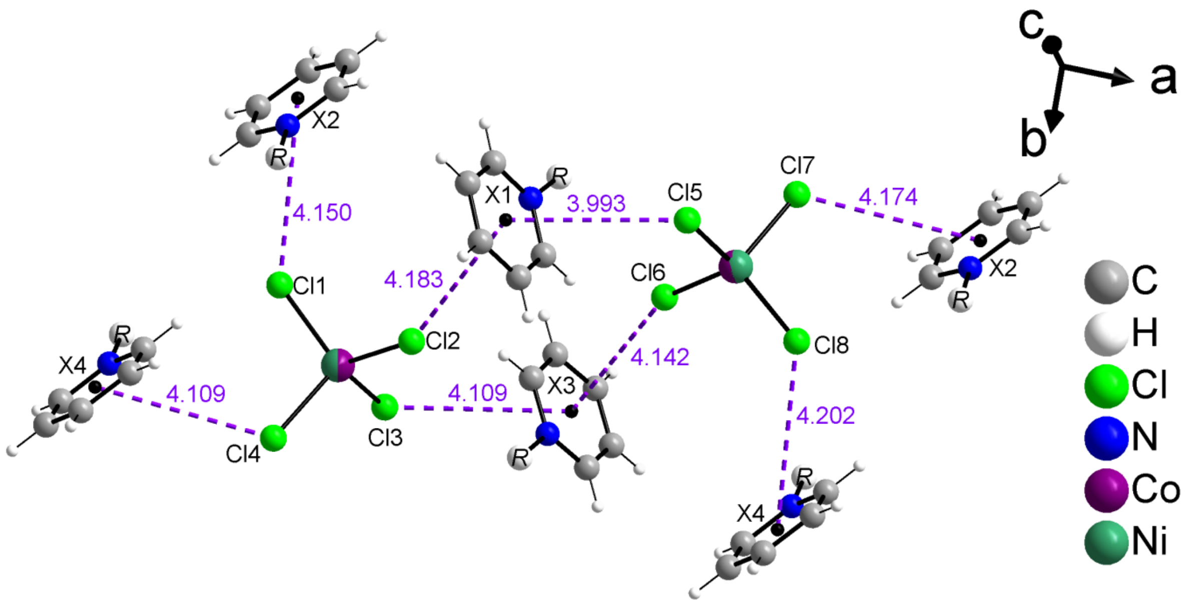

Figure S9: Anion-π interactions between pyridine rings of the cations and chloride ligands of the anions in **3**. Different aromatic centers are labeled as X1 to X4, the butyl substituents of the cations are omitted and shown as R.

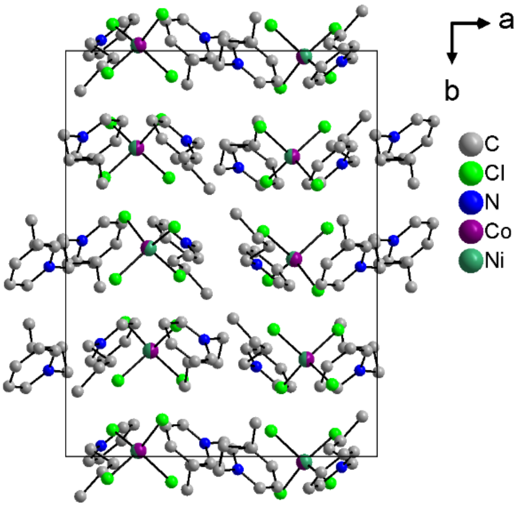

Figure S10: Packing of the compound **3** with view along the c axis (hydrogen atoms were omitted).

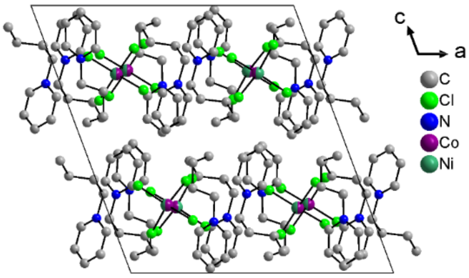

Figure S11: Packing of the compound **3** with view along the b axis (hydrogen atoms were omitted).

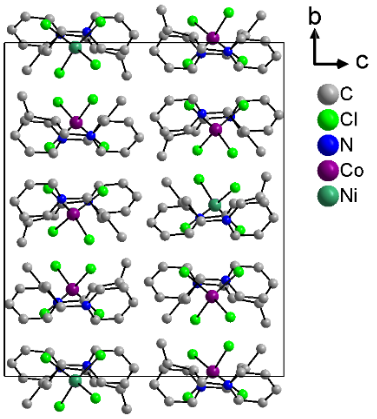

Figure S12: Packing of the compound **3** with view along the a axis (hydrogen atoms were omitted).

Compound 7:

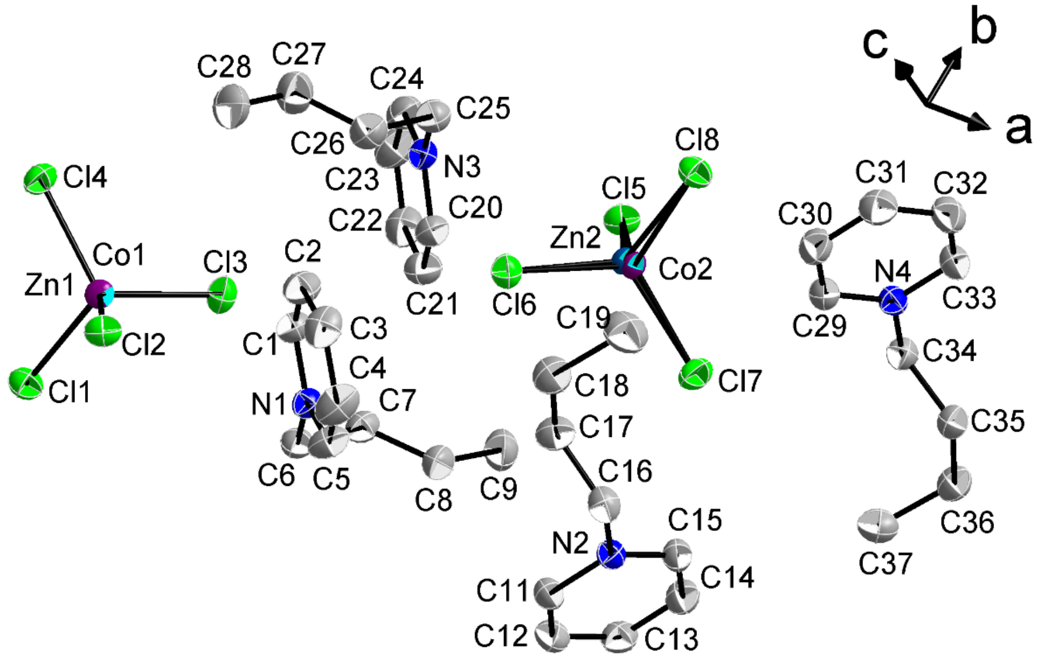

Figure S13: Structure of **7** with atomic labels, the mixed occupation of Zn and Co is shown, hydrogen atoms were omitted. Displacement ellipsoids are shown at the 50% probability level.

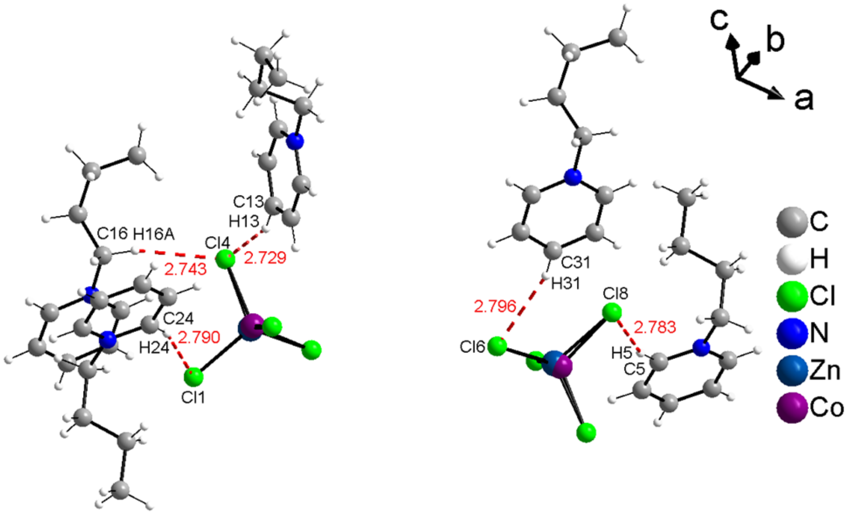

Figure S14: Hydrogen bonds between the cations and the anions in **7**. Only hydrogen bonds with a hydrogen-acceptor distance up to 2.8 Å are shown.

Table S3: Geometrical data for the intermolecular hydrogen bonds of $(C_4Py)_2[Zn_{0.54}Co_{0.46}Cl_4]$ **7**.

D—H···*A*	*D*—H	H···*A*	*D*···*A*	*D*—H···*A*
C1—H1···Cl3	0.94	2.93	3.788 (2)	153
C2—H2···Cl1^{i}	0.94	2.88	3.571 (2)	131
C4—H4···Cl5^{ii}	0.94	2.98	3.644 (2)	129
C5—H5···Cl8^{ii}	0.94	2.78	3.688 (2)	162
C6—H6*A*···Cl8^{ii}	0.96 (2)	2.98 (2)	3.866 (2)	154.8 (16)
C6—H6*B*···Cl3	0.96 (2)	2.91 (2)	3.613 (2)	130.2 (14)
C11—H11···Cl5^{ii}	0.94	2.87	3.7098 (19)	149
C11—H11···Cl8^{ii}	0.94	2.90	3.5871 (19)	131
C14—H14···Cl2^{iii}	0.94	2.90	3.5621 (19)	129
C15—H15···Cl4^{iv}	0.94	2.94	3.7622 (19)	148
C16—H16*A*···Cl4^{iv}	0.98	2.74	3.693 (2)	165
C16—H16*B*···Cl5^{ii}	0.98	2.84	3.627 (2)	138
C20—H20···Cl6	0.94	2.89	3.750 (2)	153
C23—H23···Cl2^{v}	0.94	2.98	3.645 (2)	129
C24—H24···Cl1^{v}	0.94	2.79	3.695 (2)	161
C25—H25*A*···Cl1^{v}	0.98	2.96	3.855 (2)	152
C25—H25*B*···Cl6	0.98	2.93	3.618 (2)	128
C29—H29···Cl7	0.94	2.97	3.795 (2)	147
C30—H30···Cl8^{vi}	0.94	2.88	3.574 (2)	131
C33—H33···Cl1^{vii}	0.94	2.90	3.5802 (19)	130
C33—H33···Cl2^{vii}	0.94	2.81	3.6125 (19)	143
C34—H34*A*···Cl7	0.98 (2)	2.80 (2)	3.741 (2)	163.4 (16)
C34—H34*B*···Cl2^{vii}	1.01 (2)	2.85 (2)	3.634 (2)	135.5 (14)
C35—H35*B*···Cl4^{iv}	0.94 (2)	2.90 (2)	3.741 (2)	149.3 (16)

Symmetry codes: (i) *x*+1/2, −*y*+1/2, *z*+1/2; (ii) −*x*+3/2, *y*−1/2, −*z*+3/2; (iii) *x*+1/2, −*y*+1/2, *z*−1/2; (iv) *x*+1, *y*, *z*; (v) −*x*+1/2, *y*+1/2, −*z*+3/2; (vi) −*x*+2, −*y*+1, −*z*+2; (vii) −*x*+3/2, *y*+1/2, −*z*+3/2

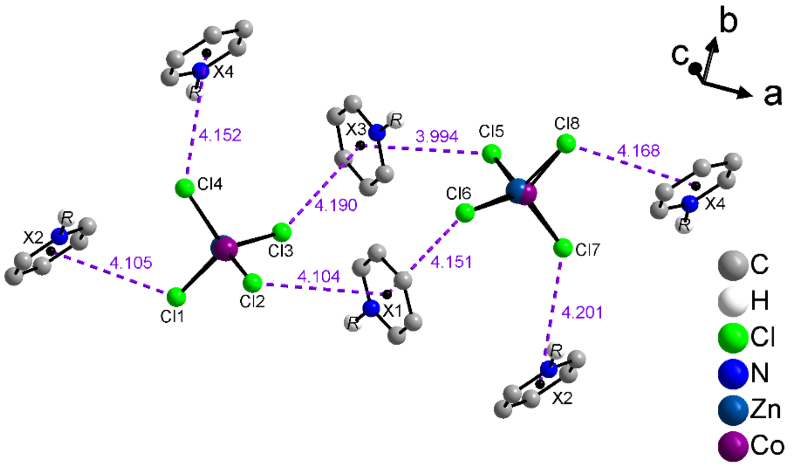

Figure S15: Anion-π interactions between pyridine rings of the cations and chloride ligands of the anions in **7**. Different aromatic centers are labeled as X1 to X4, the butyl substituents of the cations are omitted and shown as R.

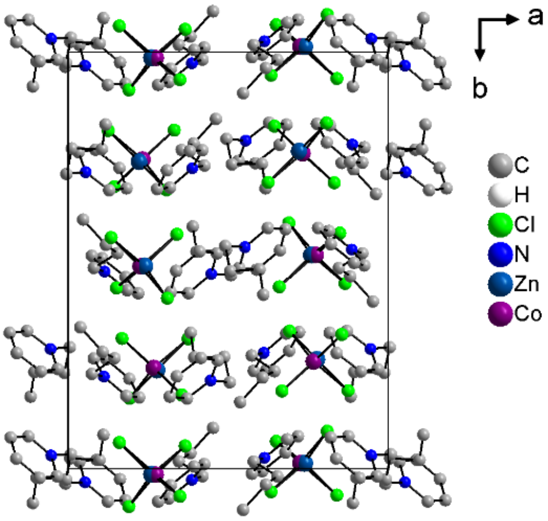

Figure S16: Packing of the compound **7** with view along the c axis (hydrogen atoms were omitted).

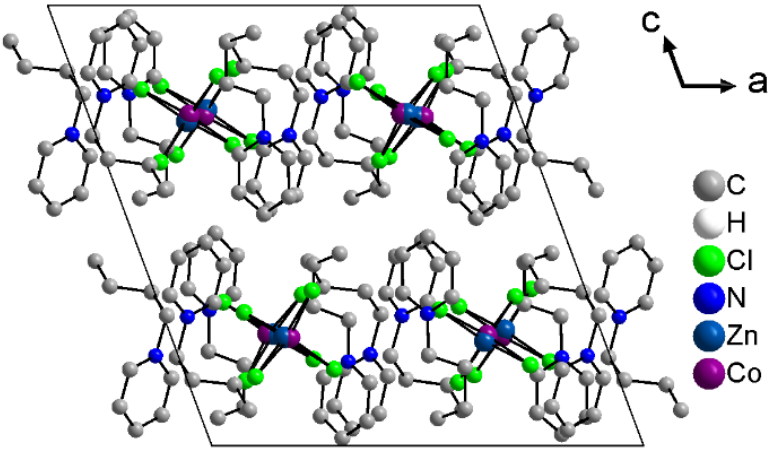

Figure S17: Packing of the compound **7** with view along the b axis (hydrogen atoms were omitted).

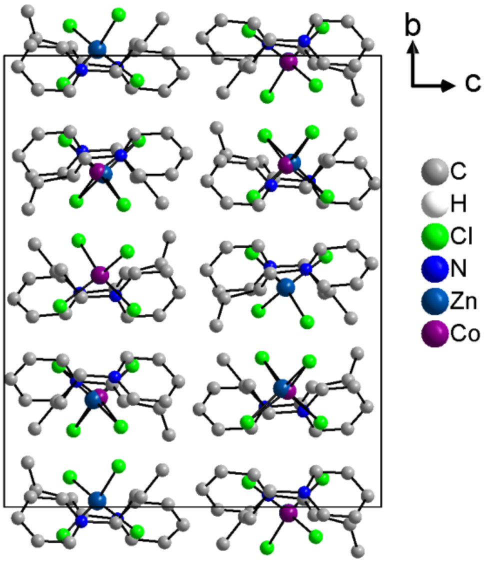

Figure S18: Packing of the compound **7** with view along the a axis (hydrogen atoms were omitted).

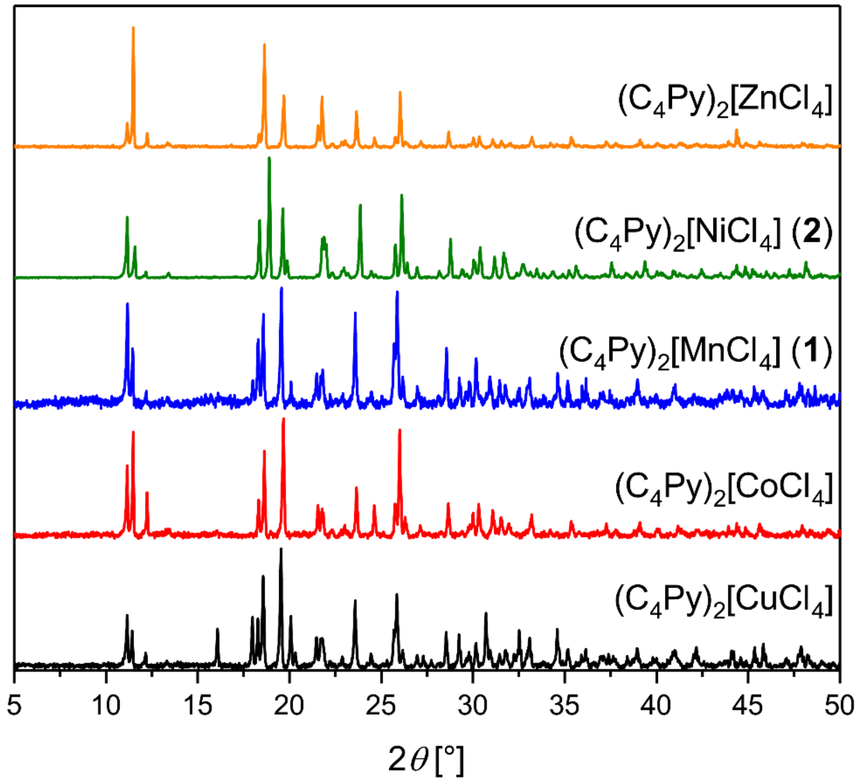

Figure S19. Powder XRD data obtained from ILs with only one metal. Data are shifted vertically to show all spectra clearly; y-axis shows X-ray reflection intensity (X-ray counts, a.u.).

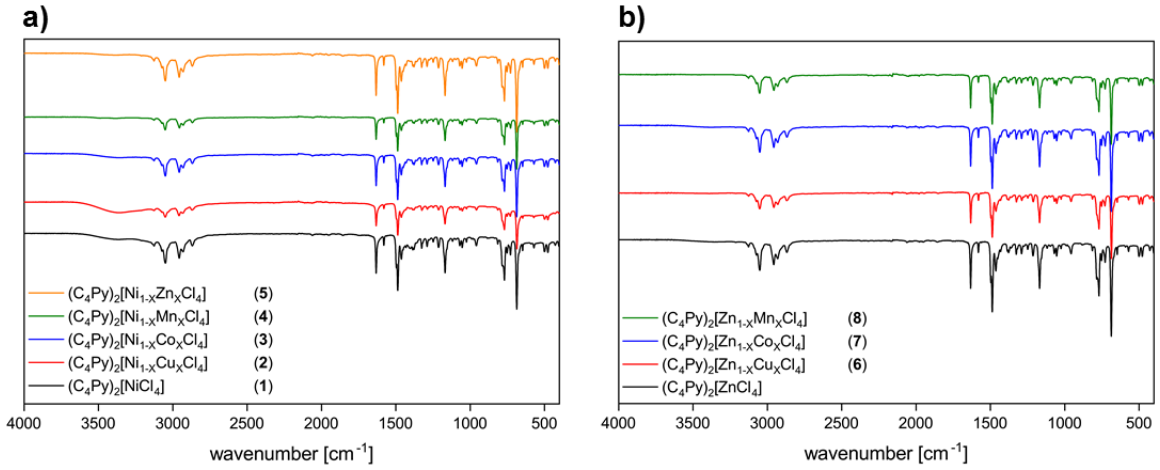

Figure S20. IR spectra of ILs **1** - **8**. Data are shifted vertically to show all spectra clearly; y-axis shows transmission.

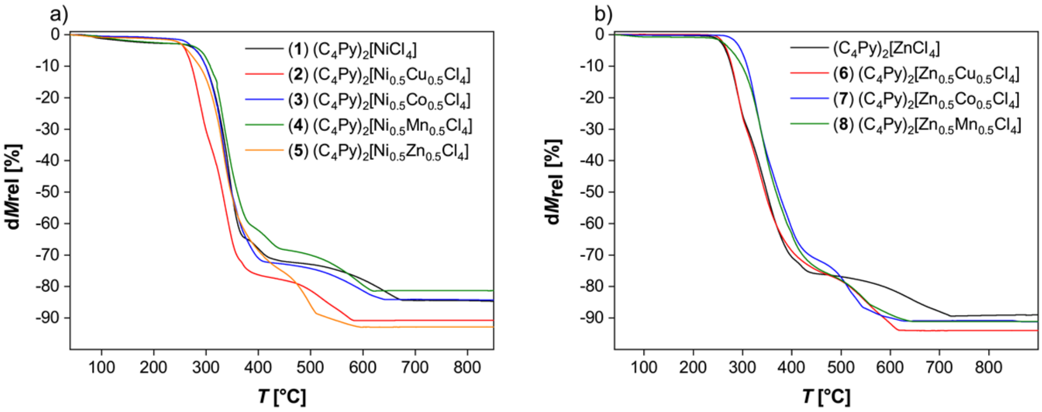

Figure S21. TGA data of ILs **1** to **8**.

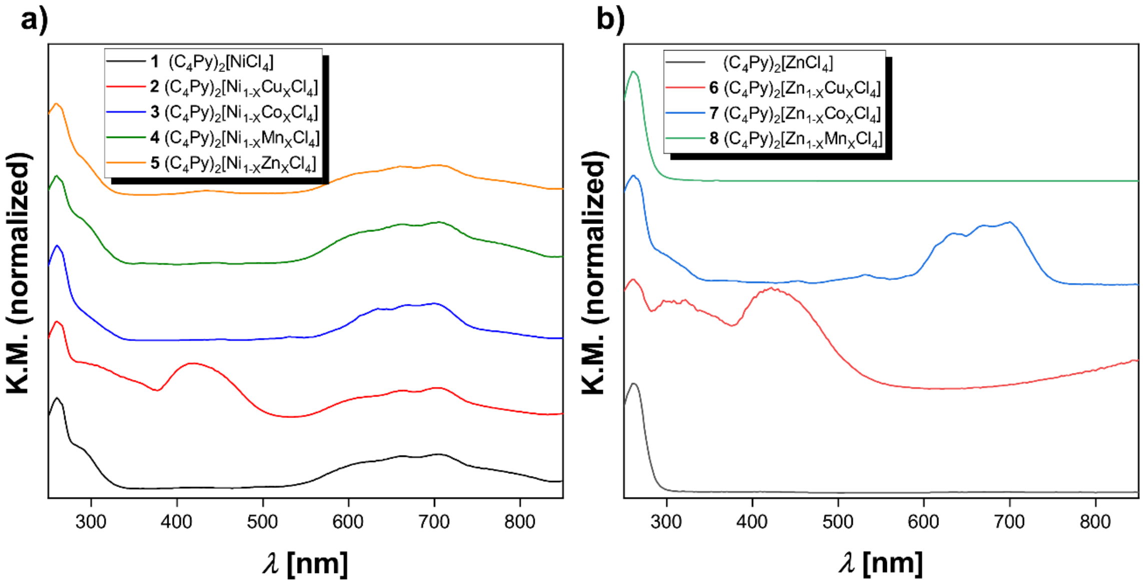

Figure S22. UV Vis spectra of ILs **1** to **8** along with spectrum of $(C_4Py)_2[ZnCl_4]$. Data are shifted vertically to show all spectra clearly; y-axis shows absorption.

In Figure S23a, the spectrum of the ionic liquid $(C_4Py)_2[Zn_{0.5}Cu_{0.5}Cl_4]$ shows a high intensity peak at 428 nm (peak 1). When the sample is exposed to ammonia, the intensity of the peak decreases dramatically and almost disappears. At the same time, a peak at 622 nm (peak 2) becomes visible. These observations could be explained in several ways: Either the peak appears as a response to ammonia or the peak overlapped with peak 1 due to its strong intensity it only became visible due to the loss of intensity of peak 1 (this can be deduced from the initial shape of the spectrum, which shows a long shoulder next to peak 1 (t = 4 min)). During the desorption phase with N2, the intensity of peak 1 increases again to almost 50%, and peak 2 experiences a red shift of almost 200 nm.

When liquid $(C_4Py)_2[Zn_{0.5}Cu_{0.5}Cl_4]$ is exposed to moist nitrogen (Figure S23b), the main peak decreases in intensity but does not disappear within the time it is exposed to moist nitrogen. Moreover, the intensity of the shoulder increases in the range from 500 nm to almost 1000 nm. Even though it could be considered as peak 2, its width seems to consist of several folded peaks. In the desorption phase, the intensity of peak 1 does not increase again, while the broad shoulder to its right disappears.

In Figure S23c, the spectrum of the ionic liquid $(C_4Py)2[Cu_{0.5}Ni_{0.5}Cl_4]$ first shows a flat peak at 432 nm (peak 1) and a less intense broad peak at 680 nm (peak 2). Upon exposure to ammonia, peak 1 loses intensity and peak 2 shifts to the shorter wavelength region by about 60 nm. During the desorption phase and for the first few minutes (t = 13 min), peak 1 recovers its intensity and becomes a sharper peak, while peak 2 appears to merge with another peak that appears to be between 800 nm and 1000 nm. After another recovery period, the intensity of peak 1 increases significantly, while peak 2 is again detectable, but at a lower intensity than at the beginning of the analysis.

In Figure S23d, the ionic liquid $(C_4Py)2[Cu_{0.5}Ni_{0.5}Cl_4]$ is exposed to wet N_2. Peak 1 changes its shape to a sharper form and its maximum shifts to the shorter wavelength region. Meanwhile, peak 2 disappears and a broad band becomes visible at 900 nm. It is imprudent to say that peak 2 has shifted to the longer wavelength region, since one peak disappears and the other suddenly appears after only 6 seconds of exposure to wet N_2. In the desorption phase, peak 1 increased in intensity as in the ammonia exposure, while peak 2 returns almost completely to its initial position and has almost the same intensity.

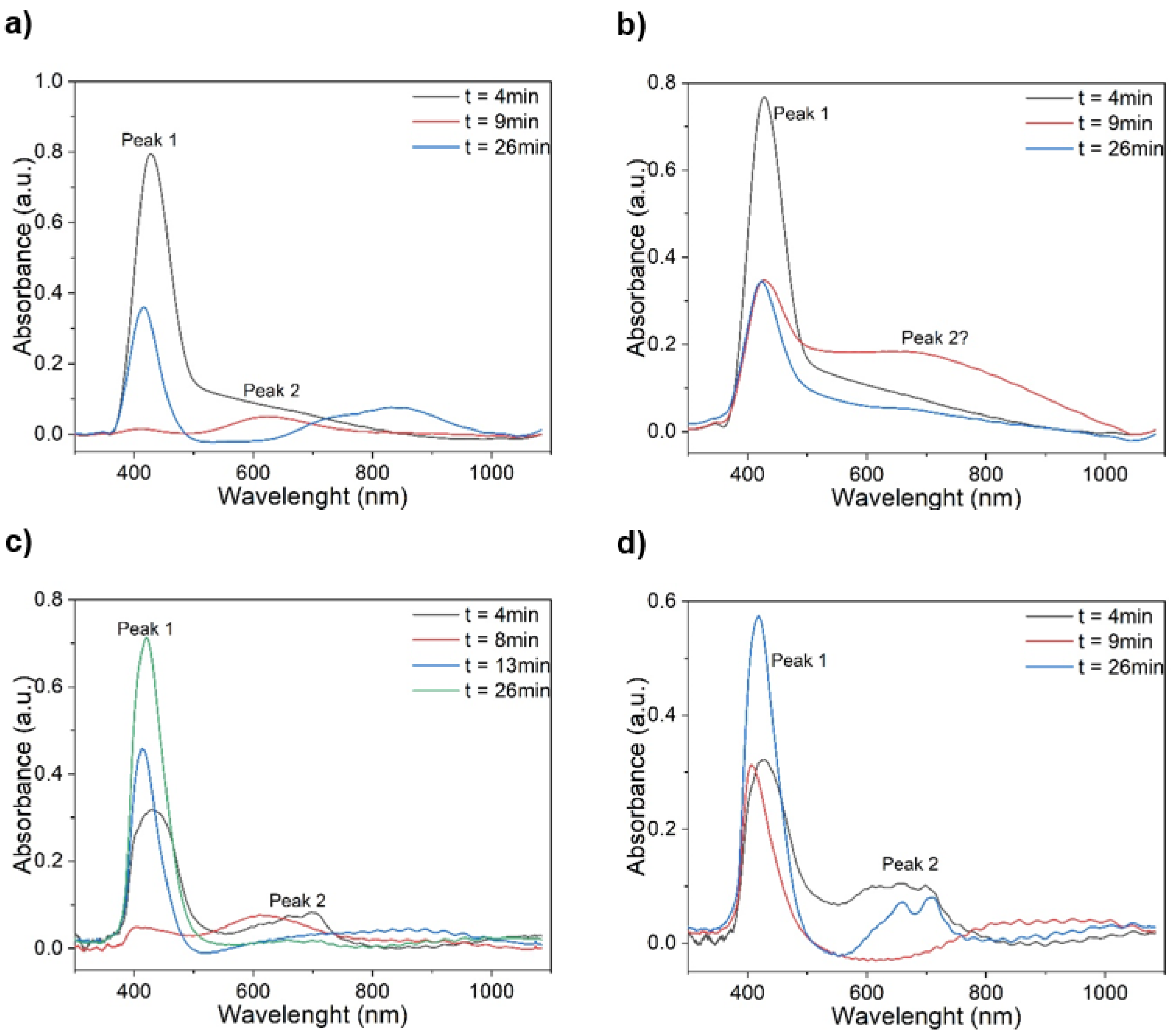

Figure S23. Analysis of absorption spectra of thin films of $(C_4Py)_2[Zn_{0.5}Cu_{0.5}Cl_4]$ and $(C_4Py)_2[Cu_{0.5}Ni_{0.5}Cl_4]$ on glass used as a gas sensor. The description of the sensor experiment is as follows: From minute 0 to 5 flow of dry N_2, from minute 5 to 10 flow of a mixture of nitrogen and ammonia or of nitrogen and water, and from minute 10 to 29 flow of dry N_2. Items (a) and (b) refer to $(C_4Py)_2[Zn_{0.5}Cu_{0.5}Cl_4]$ and items (c) and (d) refer to $(C_4Py)_2[Cu_{0.5}Ni_{0.5}Cl_4]$. The experiments in (a) and (c) were performed with ammonia containing nitrogen, and the experiments in (b) and (d) were performed with wet N_2.

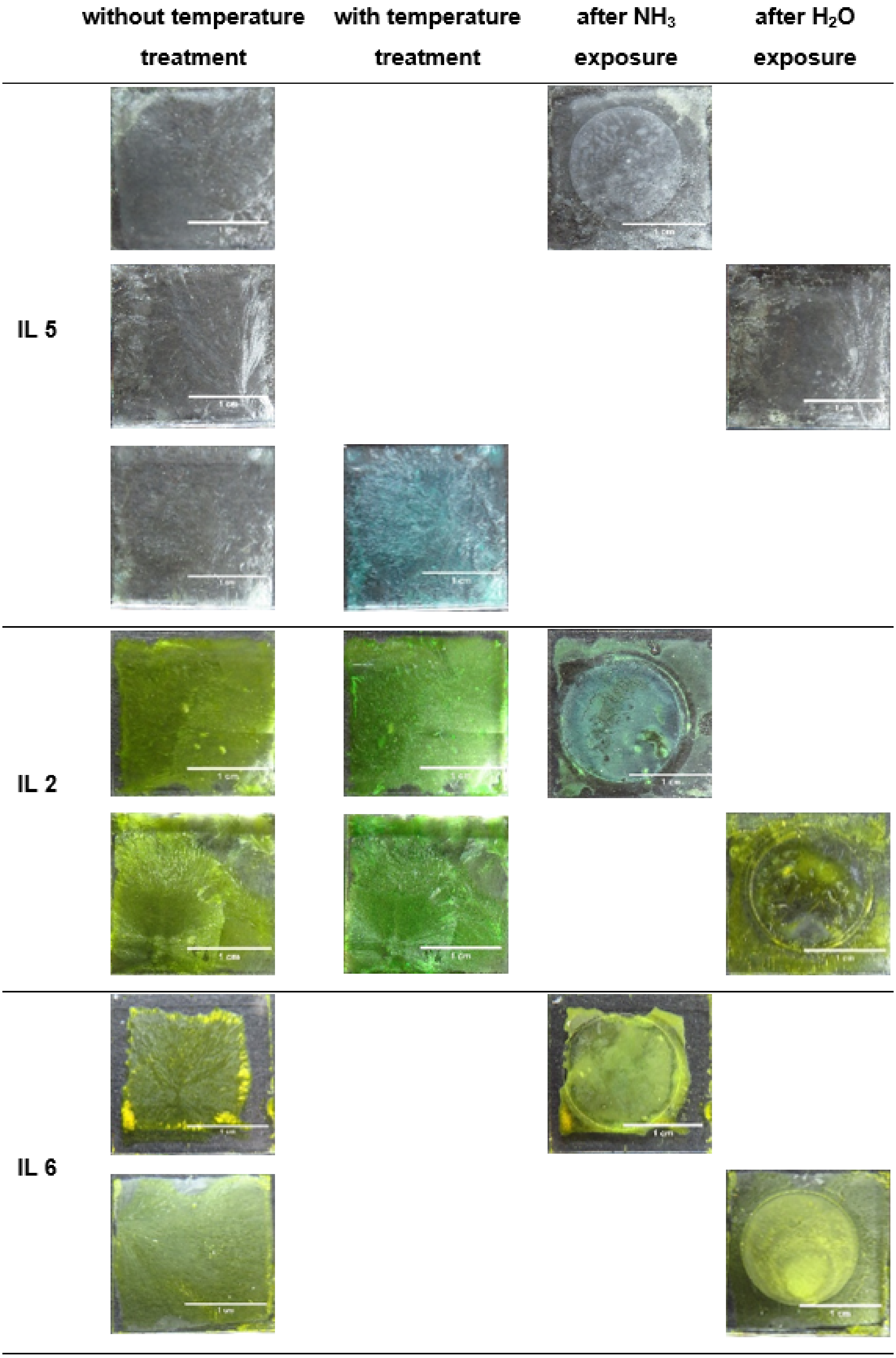

Figure S24. Photographs of the samples, before and after temperature treatment and after NH_3 respective H_2O exposure. Scale bars: 1 cm?

References

1. Steiner, T. Lengthening of the Covalent X−H Bond in Heteronuclear Hydrogen Bonds Quantified from Organic and Organometallic Neutron Crystal Structures. *J. Phys. Chem. A* **102**, 7041–7052 (1998).

2. Steiner, T. & Desiraju, G. R. Distinction between the weak hydrogen bond and the van der Waals interaction. *Chem. Commun.* **0**, 891–892 (1998).

3. Steiner, T. *et al.* Very long C–H···O contacts can be weak hydrogen bonds: experimental evidence from crystalline [Cr(CO)3{η6-[7-exo-(C□CH)C7H7]}]. *Chem. Commun.* **1**, 171–172 (1998).

4. Frontera, A., Gamez, P., Mascal, M., Mooibroek, T. J. & Reedijk, J. Putting Anion-π Interactions Into Perspective. *Angew. Chemie Int. Ed.* **50**, 9564–9583 (2011).

5. Balischewski, C. *et al.* Ionic liquids with more than one metal: optical and electrochemical properties vs. d-metal combinations. *Chem. – A Eur. J.* (2020) doi:10.1002/chem.202003097.

6. Balischewski, C. *et al.* Tetrahalidometallate(II) ionic liquids with more than one metal: the effect of bromide vs. chloride. *Chem. – A Eur. J.* (2022) doi:10.1002/chem.202201068.
